# Cryptic species in the parasitic *Amoebophrya* species complex revealed by a polyphasic approach

**DOI:** 10.1038/s41598-020-59524-z

**Published:** 2020-02-13

**Authors:** Ruibo Cai, Ehsan Kayal, Catharina Alves-de-Souza, Estelle Bigeard, Erwan Corre, Christian Jeanthon, Dominique Marie, Betina M. Porcel, Raffaele Siano, Jeremy Szymczak, Matthias Wolf, Laure Guillou

**Affiliations:** 1 Sorbonne Université, CNRS, UMR7144 Adaptation et Diversité en Milieu Marin, Ecology of Marine Plankton (ECOMAP), Station Biologique de Roscoff SBR, 29680 Roscoff, France; 2 Sorbonne Université, CNRS, FR2424 ABIMS, Station Biologique de Roscoff SBR, 29680 Roscoff, France; 3 Algal Resources Collection, MARBIONC, Center for Marine Sciences, University of North Carolina Wilmington, 5600 Marvin K. Moss Lane, Wilmington, NC 28409 US; 4 Génomique Métabolique, Genoscope, Institut François Jacob, CEA, CNRS, University Evry, Université Paris-Saclay, 91057 Evry, France; 5 Ifremer-Centre de Bretagne, Département/Unité/Laboratoire ODE/DYNECO/Pelagos, Z.I. Technopôle Brest-Iroise, Pointe du Diable BP70, 29280 Plouzané, France; 6 Department of Bioinformatics, Biocenter, University of Würzburg, Am Hubland, 97074 Würzburg Germany

**Keywords:** Microbial ecology, Parasitology

## Abstract

As critical primary producers and recyclers of organic matter, the diversity of marine protists has been extensively explored by high-throughput barcode sequencing. However, classification of short metabarcoding sequences into traditional taxonomic units is not trivial, especially for lineages mainly known by their genetic fingerprints. This is the case for the widespread *Amoebophrya ceratii* species complex, parasites of their dinoflagellate congeners. We used genetic and phenotypic characters, applied to 119 *Amoebophrya* individuals sampled from the same geographic area, to construct practical guidelines for species delineation that could be applied in DNA/RNA based diversity analyses. Based on the internal transcribed spacer (ITS) regions, ITS2 compensatory base changes (CBC) and genome *k*-mer comparisons, we unambiguously defined eight cryptic species among closely related ribotypes that differed by less than 97% sequence identity in their SSU rDNA. We then followed the genetic signatures of these parasitic species during a three-year survey of *Alexandrium minutum* blooms. We showed that these cryptic *Amoebophrya* species co-occurred and shared the same ecological niche. We also observed a maximal ecological fitness for parasites having narrow to intermediate host ranges, reflecting a high cost for infecting a broader host range. This study suggests that a complete taxonomic revision of these parasitic dinoflagellates is long overdue to understand their diversity and ecological role in the marine plankton.

## Introduction

The accurate estimation of the diversity of protists (i.e., eukaryotic microbes) is crucial for gaining a better understanding of their ecological roles in the world oceans^1,2^. However, traditional morphology-based methods for species delineation are challenging to apply to single-cell organisms where morphological features are frequently not discriminative enough, with few alternatives explored so far^3,4^. The inventory of the planktonic protist diversity in marine systems has recently expanded thanks to culture-independent, DNA barcode-based methods directly applied in the field over large geographic scales^5,6^. While this avalanche of environmental sequences is generally classified into manageable operational taxonomical units (OTUs), the correct assessment of the quantitative contribution and functional roles of marine pelagic protists is, however, hindered by the uncertainty of real species richness. In other words, intraspecific sequence variation within morphospecies needs to be differentiated from “true” species diversity^7^. So far, there are no universal rules linking molecular data to species richness in marine protists, partially due to the low incidence of observed sexual recombination, morphological and evolutionary convergence, and sometimes high discordance between genetic and phenotypic characters^8^.

Parasitism is an essential ecological process contributing to the resilience of ecosystems while acting as an evolutionary pressure for both hosts and parasites^9^. Due to the high diversity and ubiquity of parasites, understanding the factors that generate, maintain, and constrain host-parasite interactions is of primary interest in ecology and evolution. Thus, achieving a reliable delineation of cryptic species within parasitic protistan lineages is critical for gaining a better knowledge of their ecological niches and host range. The problem of species delineation is pervasive for parasitic lineages almost exclusively composed of environmental sequences, such as the Marine ALVeolate lineages (MALVs)^10,11^. MALV represented one of the most hyperdiverse lineages (>1,000 OTUs) recovered in the metabarcoding dataset collected during the Tara Oceans expedition^5,12^. However, only a handful of species representatives of the different MALV lineages have been formally described, all of them obligatory aplastidial parasites occurring as intracellular biotrophs (i.e., the host is maintained alive during the infection but eventually killed) and belonging to the order Syndiniales^11^. Among them, Amoebophryidae (or MALV-II) were observed to have the highest rate of cladogenesis (i.e., speciation minus extinction rates) among 65 marine protist lineages^13^, making their classification even more challenging.

The *Amoebophrya ceratii* species complex is a MALV-II clade with a worldwide distribution that can be isolated in culture^14,15^. All *A. ceratii* reported to date were observed infecting a broad range of marine dinoflagellates^11,16^. A single infected host produces within days hundreds of dinospores (i.e., free-living, flagellated infective propagules), each with a life span of few days^17^. Dinospores frequently account for a substantial proportion (>25%) of the nanoplanktonic fraction (2–20 µm) in coastal waters^18^ and can be readily consumed by microzooplankton grazers (20–200 µm)^19^. Consequently, such parasites potentially constitute key trophic links between different compartments of the marine food web in the oceanic carbon cycle^20^, notably through population control of dinoflagellate blooms^21,22^.

Here, we explored the diversity of the *A. ceratii* species complex through an extensive sequencing effort of 76 strains in culture and 43 environmental single-cells from two close localities (the Penzé and Rance Estuaries, western Channel, France). We followed a polyphasic approach to provide the first comprehensive species boundaries delineation within the *A. ceratii* species complex. To do so, we combined (i) ribotyping (both of the SSU rDNA and ITS1-5.8S-ITS2 regions), (ii) *k*-mer analysis from whole-genome sequencing, (iii) analysis of the ITS2 compensatory base changes (CBCs), (iv) phenotypic characteristics of dinospores by flow cytometry, and (v) assessment of their host range through cross-infection culture experiments. Finally, we applied our novel species boundaries to answer the following questions: do these *Amoebophrya* cryptic species share the same ecological niches? Can we explain their fitness (maximal abundance and persistence in time) by their host range? For that, we explored the population dynamics of the newly-defined cryptic *Amoebophrya* species (considered here as ribotypes until formal descriptions are performed) during a three-year survey of the toxic dinoflagellate *Alexandrium minutum* in the Penzé Estuary, a site and a period of the year previously reported to have high diversity of *Amoebophrya* ribotypes infecting a wide range of dinoflagellate species with prevalences as high as 40% of the total host abundance^21^. This study constitutes the first evaluation of the interannual variability of *Amoebophrya* species, their ecological niches, and population fitness in the field.

## Materials and Methods

### Origin of *Amoebophrya* strains and single infected dinoflagellate cells

We based our analyses either on *Amoebophrya* strains or directly on infected host cells isolated by micromanipulation from environmental samples (hereafter called single-cells). Strains and single-cells were isolated during monitoring for the toxic dinoflagellate species *Alexandrium minutum*. Monitoring was performed over five years (2007, 2009, 2010–2012) in the Penzé Estuary (48°37′37.57″N, 3°57′13.17″W) and in 2011 in the Rance Estuary (48°31′49.61″N, 1°58′21.81″W), both located in the western Channel (France). Sampling started before the *A. minutum* bloom (late May-early June) and stopped at the end of the bloom (end of June, beginning of July), generally after 5–7 weeks. Planktonic communities were collected every 1–2 days. For biotic parameters, we fixed cells (>10 µm) with Lugol’s solution and used flow cytometry to count bacteria, viruses, cyanobacteria, picoeukaryotes and phototrophic cryptophytes (based on their pigment and DNA contents). We recorded abiotic parameters including salinity, temperature (air and water), nutrient concentrations (NO_3_, NH_4_, and PO_4_), rainfall and light intensity. Detailed information on the sampling strategy and data acquisition can be found in previously published data focusing on *A. minutum* blooms^9,21,23^.

For single-cells, host cells in the late stages of infection by *Amoebophrya*-like parasites were detected from freshly collected field samples (less than 3 hours) through their natural green autofluorescence using an epifluorescence microscope (BX51, Olympus) equipped with the U-MWB2 cube (450- to 480-nm excitation, 500-nm emission^24^), then sorted individually by micropipeting, and washed three times into filter-sterilized (<0.22 µm) freshly prepared medium. Hosts were identified according to their morphology, and the single cells were transferred into cryovials with a minimum of medium (3–5 µl), flash-frozen, and stored at −80 °C. DNA extraction and purification were performed both on pelleted strains and single-cells using the MasterPure kit (Epicentre).

To culture *Amoebophrya* strains, our strategy was to isolate representative phototrophic dinoflagellates, as potential hosts, from the Rance and Penzé Estuaries and other estuaries nearby. We initiated infections in the dinoflagellates by *Amoebophrya*, either using 3–5 µm filtered samples (fraction presumably containing dinospores) and single, infected dinoflagellate cells (isolated as explained above). *Amoebophrya* strains were kept in their initial hosts until we reduced the number of hosts to facilitate their maintenance (using either *Heterocapsa triquetra* or *Scrippsiella acuminata* STR1). Additional details regarding the isolation and maintenance of strains are described in the supplementary information.

### Genome sequencing

Our strategy to discriminate individuals (i.e., strains and single-cells) was to find fundamental units that formed separate branches on rRNA phylogenetic trees (i.e., ribotypes) and then check whether these fundamental units (or clades) shared a unique combination of phenotypic characters as the backbone for their taxonomy. For that, strains and single-cells were screened by sequencing the ITS1-5.8S-ITS2 region of the ribosomal operon, as explained in Blanquart *et al*.^9^. Then, Illumina whole-genome sequencing was performed on a selection of 50 cultivated strains (where the flow cytometry-estimated bacterial contamination was <10%) and 17 single-cells in order to maximize the number of representative *Amoebophrya* ribotypes. The methodology for cell harvesting for genomic analysis is detailed in the protocole.io dx.doi.org/10.17504/protocols.io.vrye57w. Whole-genome amplification from single-cells was performed using a multiple displacement amplification (MDA) approach with RepliG (QIAGEN, Courtaboeuf, France) according to the manufacturer’s instructions. Paired-end libraries were prepared individually and sequenced on an Illumina HiSeq2000 platform, and a draft genome was assembled for each of the strains. More details regarding sequencing and genome assembly are described in the supplementary information.

### Ribosomal operons analyses

We estimated the average number of ribosomal operons per *Amoebophrya* genome by comparing the read coverage to that of a list of putatively single-copy genes (initial list of 67 genes) (unpublished data). To do so, we first used a BLASTn (e-value < 0.0001) search against the draft genome assemblies to capture the ribosomal operon and the genes of interest. A gene was discarded from the putative single-copy gene list either if i) it was detected in multiple copies using a reciprocal BLAST approach, or ii) had no hit. Genomic reads were then mapped to each of the best hits using Bowtie2^25^. Only the aligned region (i.e., high-scoring pairings as reported by BLASTn) was used for calculating the average coverage of the reference genes and then used to estimate the number of repeated ribosomal operons per genome. In doing so, we used an average of 21 genes per strain (minimum 7; maximum 55).

### Compensatory base changes (CBCs)

Full-length ITS2 sequences were directly annotated using Hidden Markov Models (HMMs)^26^ as implemented in the ITS2 database^27^ or by alignment to annotated sequences. Secondary structures were predicted by homology modeling using a relevant template (e.g.^26,27^, or by RNA structure using energy minimization and constraint folding^28,29^. The phylogenetic analysis of the ITS2 dataset followed the procedures outlined in^30^. Specifically, a global multiple sequence-structural alignment was automatically generated in 4SALE v1.7^30–32^, whereby ITS2 sequences and their respective secondary structures were simultaneously aligned using a 12 × 12 ITS2 sequence-structure specific scoring-matrix^33^. Phylogenetic relationships were reconstructed by neighbor-joining (NJ) through the use of an ITS2 sequence-structure specific Jukes-Cantor correction (JC) or an ITS2 sequence-structure specific general time-reversible (GTR) substitution model, both implemented in ProfDistS v0.9.9^34^. Using the ITS2 sequence and secondary structure simultaneously (encoded by a 12-letter alphabet^33^,), a maximum parsimony tree (MP) was reconstructed by PAUP^35^ based on default settings. A sequence-structure maximum likelihood tree (ML) was calculated using the “phangorn” package^36^ in R^37^. Bootstrap support was estimated from 100 replicates. A CBC table was transferred from 4SALE^32^.

### Genome comparison using SIMKA *k*-mer analysis

We estimated the *k*-mer distribution of genomes using SIMKA (*k* = 21 bp; minimum read size ≥90 bp, Shannon index <1.5)^38^. Due to inherent differences in the genome coverage obtained from cultivated strains and single-cells, we based the cluster analysis upon the presence/absence of *k*-mers by considering only the distance indexes (based on the formulas given by^38^) that give more weight to the double presence of *k*-mers (i.e., Kulczynski, Ochiai, and Chord/Hellinger distances)^39^. Bootstrap analysis after 100 permutations were obtained using the *clusterboot* function from the ‘fpc’ R package, directly performed on the distance matrix output by SIMKA with ‘clusterCBI’ as the clustering method, considering the above-estimated number of ribotypes as the desired number of clusters.

### Cell phenotype

The rationale for not using morphology and ultrastructure for the characterization of these strains can be found in the supplementary information. Phenotypic characteristics of the strains were deduced from their flow cytometric signatures [i.e., side scatter (SSC), forward scatter (FSC), and natural green autofluorescence], by directly loading 500 µl of fresh cultures on a FACsAria flow cytometer (Becton Dickinson, New Jersey, USA). We additionally estimated the genome size of each strain following the procedure explained in^40^, where the ratio between the mean distribution of the dinospores and the internal reference *Micromonas pusilla* RCC299 cells (1 C = 20.9 fg) was used for the evaluation of the nuclear DNA content.

### Host range

We determined the host range of the *Amoebophrya* strains through cross-infection experiments using a diverse selection of locally-occurring dinoflagellate strains isolated from the Rance and Penzé Estuaries and nearby estuarine systems (Table S1, Fig. S2). Freshly produced dinospores were collected by filtration through 5-µm pore-sized cellulose acetate filters (Minisart, Sartorius, Germany) and 100 µl aliquots of this filtrate were inoculated into 1 ml of exponentially growing dinoflagellate strains into 24-well plates. Infections by *Amoebophrya* strains were detected based on their natural green fluorescence after 2–5 days. Hosts were classified either as resistant (no trace of infection) or sensitive (at least one infected host cell). All cross-infections were processed 3–5 times at different dates.

### Environmental metabarcoding survey

We obtained environmental rDNA metabarcoding sequences of 48 samples of the >10-μm size-fraction collected in the Penzé Estuary during late spring and early summer between 2010 and 2012. The DNA was extracted using the phenol-chloroform protocol^41^, followed by the amplification of the SSU rDNA V4 region (~380 bp) using the universal forward TAReuk454FWD1 primer (5′-CCAGCASCYGCGGTAATTCC-3′), and the modified reverse BioMarKs primer (5′-ACTTTCGTTCTTGATYRATGA-3′)^42^. PCR amplifications were performed in duplicates for each sample using 5 μM of each primer, 5 μl of 5x buffer, 37.5 mM of magnesium chloride, 6.25 mM of dNTPs, 0.5 unit of GoTaq Flexi (Promega, Wisconsin, USA), approximately 2 ng of DNA (25 μl final volume) and the PCR cycles (initial denaturation: 95 °C for 3 min, 22 to 25 cycles: 95 °C for 45 s, 50 °C for 45 s, 68 °C for 90 s, and final extension: 68 °C for 5 min). The GeT-PlaGe platform (Toulouse, France) performed the Illumina Miseq library preparation and the paired-end sequencing. Taxonomic annotations were performed on unique sequences (100% threshold sequences similarity) observed in at least two different libraries using Mothur^43^ implemented by the PR2 reference database^44^ modified to take into account the different ribotypes of *Amoebophrya* recognized in this study.

### Statistical analyses

All the statistical analyses described below were performed in R software using packages freely available on the CRAN repository (http://www.cran-r-project.org).

#### Comparison of ribotypes based on flow cytometry features, number of operons and host range

We first used Pearson correlations to establish whether the different morphological variables monitored here (excluding host range) were related to one another. Then, differences between ribotypes were assessed by pairwise Mann-Whitney analysis using the *cor.test* and *wilcox.test* functions from the basic ‘stats’ package based on [log (*x* + 1)] transformed data. For comparison of *Amoebophrya* ribotypes based on their host range, results from the cross-infections were organized into a presence/absence matrix (i.e., infection = 1; no infection = 0) with parasites in the columns and dinoflagellate host strains in the rows. This matrix was then used to generate a heatmap using the function *heatmap.2* of the ‘gplots’ package^45^. Finally, we assessed the relative importance of the phenotypic characters and the host range in the differentiation of the strains belonging to the different ribotypes through a principal coordinate analysis (PCoA) using the *cmdscale* function of the ‘stats’ package. The PCoA was based on Bray-Curtis distances calculated with the ‘vegan’ package^46^ from a matrix of descriptors including the standardized values (between 0 and 1) of the phenotypical characters (estimated from their minimum and maximum values^47^), as well as the presence and absence of infections (1 and 0, respectively) in the different host species. The *envfit* function of the ‘vegan’ package was used to fit the descriptors to the two first PCoA axes.

#### Niche analysis

The Outlying Mean Index (OMI) analysis^48^ was first performed to determine the niche position and niche breadth of *Amoebophrya* ribotypes using the function *niche* in the ‘ade4’ package^49^. We included all 1,153 unique sequences detected in the metabarcodes (distributed into different phylogenetic lineages) to get a better resolution in the niche position of the *Amoebophrya* ribotypes. Relative read abundances (compared to the total number of reads) and several environmental descriptors [i.e., water temperature, salinity, precipitation, tide coefficient, NO_3_, PO_4_ and Si(OH)_4_] were included in two separate matrixes (N = 48). Before analysis, relative read abundances were Hellinger transformed^50^ whereas the environmental descriptors were standardized to values between 0 and 1^47^. The function *envfit* was used to fit the environmental variables to the first two OMI axes. Sample scores from the first two OMI axes were then used to estimate the kernel density weighted by abundance^47,48^ of *Amoebophrya* ribotypes using the *kde* function from the ‘ks’ package^51^. The niche overlap was then estimated by the comparison of the realized niches (i.e., kernel densities) through the calculation of the *D* metric^52^ for each pair of *Amoebophrya* ribotypes using the *ecospat.niche.overlap* function from the ‘ecospat’ package^53^. Pair-wise *D* metrics were then used to generate a heatmap to detect clustering of the ribotypes related to their niche overlap, following the same procedure described previously for the analysis of the results of the cross-infections.

#### Relationship between the population fitness of the ribotypes and their host range

We first obtained a more precise estimate of the quantitative contribution of the different ribotypes by dividing the relative abundance of each ribotype in a given metabarcoding sample by their average number of operons estimated from the genome analysis of the strain (hereafter called “normalized abundance). We used the normalized abundances to estimate the population fitness of the six *Amoebophrya* ribotypes that could be discriminated in the metabarcodes through their V4 sequences, in each one of the three years (N = 18), based on i) their maximal normalized abundances and ii) persistence in the system (e.g., the number of consecutive days in which the non-normalized relative contribution of the ribotype to the total number of reads was higher than 10%). We then determined if these two indicators were different between groups of *Amoebophrya* ribotypes representing different host ranges (based on the maximal number of infected host species in the cross-infection experiments for each ribotype). This was assessed by Kruskal-Wallis tests using the *kruskal.test* function in the ‘stats’ package following [log (x + 1)] transformation. In the cases where the Kruskal-Wallis test was significant, the post-hoc Dunn’s test was performed with the *dunnTest* function in the ‘FSA’ package.

## Results and Discussion

### Ribotypes as cryptic species

**Figure 1:**
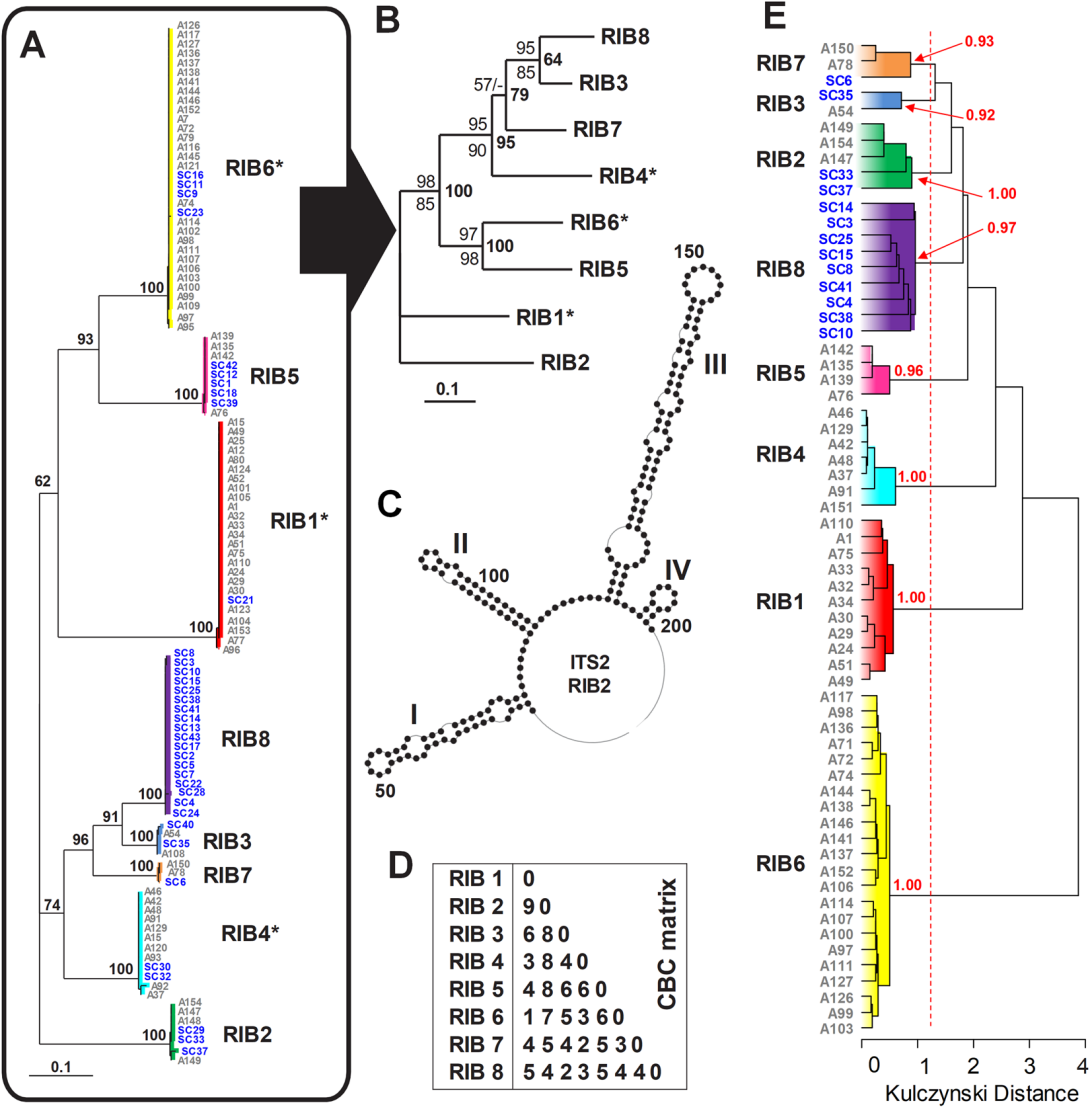
The eight *Amoebophrya* ribotypes (RIBs 1–8) defined by the ITS2 secondary structures and SIMKA *k*-mer genome comparison. (**A**) Secondary structure neighbor-joining (NJ) tree rooted with ribotype 2 (RIB2) derived from the multiple sequence-structure alignment of the ITS2 region with a 12 × 12 Jukes-Cantor correction. Bootstrap values >50 are mapped to nodes. (**B**) Secondary structure NJ tree rooted with ribotype 2 (RIB2) derived from a subset of the multiple sequence-structure alignment of the ITS2 region from (**A**) using a GTR substitution model. Bootstrap values >50 derived from NJ, maximum parsimony (MP), and maximum likelihood (ML) analyses are mapped to above, below, and to the right of the nodes, respectively. (**C**) An example of the ITS2 secondary structure from the *Amoebophrya* RIB2 clade. Helices are numbered from I to IV according to Mai and Coleman^62^. (**D**) Matrix of compensatory base changes (CBCs) between the eight *Amoebophrya* ribotypes (RIBs 1–8). (**E**) SIMKA *k*-mer genome comparison analysis based on the Kulczynski distance. Bootstrap values for terminal nodes are shown.

We amplified and sequenced part of the ITS1-5.8S-ITS2 region from 119 *Amoebophrya*-like individuals: 76 strains and 43 infected host cells isolated from environmental samples (i.e., single-cells) (Table S1). The alignment based on the secondary structure of the ITS2 region clustered individuals into eight main ribotypes (RIBs 1–8, Fig. 1A–C). We successfully isolated at least one representative in culture for each ribotype, with the notable exception of RIB8 that was only represented by environmental single-cells. Each ribotype displayed low intra-variability regarding the ITS1-5.8S-ITS2 region (i.e., <3 single-nucleotide polymorphism or SNPs) and none in the SSU rDNA region (except RIB1 contained one SNP in the V1-V2 regions). Following the nomenclature proposed by Guillou *et al*.^11^, members of RIB2 belonged to the MALV-II clade 4, whereas the remaining ribotypes were members of the MALV-II clade 2 (Fig. S1). Individuals belonging to ribotypes in MALV-II clade 2 (RIBs 1 and 3–8) shared 96–100% pairwise sequence identities, but only 93–94% with those from the RIB2 clade (Table S3). RIB3 and RIB8 were the most similar ribotypes (four SNPs in their SSU rDNA, no SNP in the V4 region and one in the V9 region; Table S3).

We investigated whether the observed rDNA sequence variability reflected species-level or intraspecific diversity by analyzing compensatory base changes (CBCs) between the ITS2 sequences in each ribotype. CBCs are mutations impacting both nucleotides of a paired region in the folded RNA transcript that maintains the pairing (e.g., A-U to G-C) and the secondary hairpin structure of the ITS2^54^. According to Müller *et al*.^55^, CBCs found in the ITS2 region of the rDNA of two seemingly-related specimens correlate (with a probability of 0.93) to the biological species concept (interbreeding populations generating fertile offspring and reproductively isolated from others) of species^56^, whereas the absence of CBC might suggest that the two ITS2 belong to the same species with a probability of 0.76. As a consequence, the CBC species concept stands as a valuable and practical alternative for indicating the potential for discriminating protistan lineages (e.g.^57–59^,). We observed no CBC within ribotypes, whereas 1–9 CBCs were observed between different ribotypes (Fig. 1D). The phylogenetically closest ribotypes RIB3 and RIB8 displayed 2 CBCs, while RIBs 1 and 6 only diverged by one CBC despite being further apart on the rDNA tree (Fig. 1A,B).

**Figure 2:**
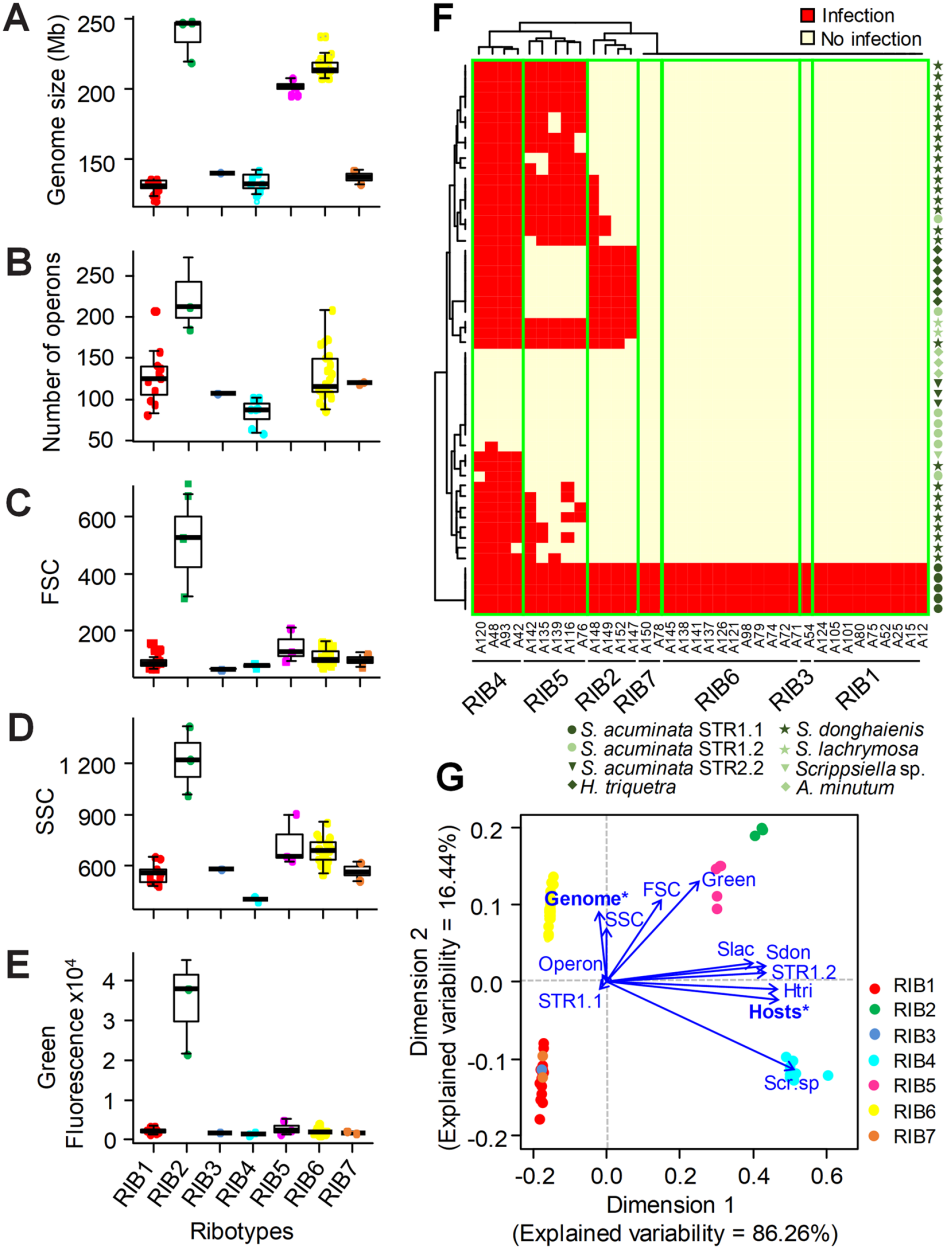
Phenotypic characters of the seven *Amoebophrya* ribotypes (RIBs 1–7) isolated in culture. (**A**–**E**) Boxplots showing predicted genome sizes (**A**), the estimated number of ribosomal operons (**B**), and flow cytometry signatures: forward scatter (FSC) (**C**), side scatter (SSC) (**D**), and green autofluorescence at 405 nm (**E**). Horizontal lines in the boxplots indicate the median values. (**F**) Heatmap showing the results of the cross-infection experiments where 36 *Amoebophrya* strains were exposed to 54 host strains distributed in 9 dinoflagellate species (see Table S2 and Figure S3 for details on the host strains). Note: RIB8 is missing because no representative for this ribotype was isolated in culture. (**G**) Ordination diagram of the principal coordinate analysis (PCoA) assessing the relative importance of six phenotypic characters (blue vectors) and host range in the differentiation of the strains belonging to the different *Amoebophrya* ribotypes. The main characters contributing to the separation of the strains (establish by the *envfit* function from the ‘vegan’ package) are indicated with asterisks. Operon = number of ribosomal operons; Green = green fluorescence; Genome = genome size; Host = maximal number of infected hosts per strain observed in the cross-infection experiments; Slac = *S. lachrymosa*; STR.1 = *S. acuminata* STR.1; Sdon = *S. donghaienis*; Htri = *H. triquetra*, Scri.sp = *Scrippsiella* sp.

Considering that CBCs and ribotypes are targeting the same genomic region (i.e., the ribosomal operon), we aimed to determine if a comparison at the genome level should be a more appropriate approach for determining species, considering that two genomes should be similar enough in size and sequence to pair during sexual reproduction. Genome sizes of strains estimated by flow cytometry oscillated between 121 and 250 Mb (Fig. 2A). Overall, we observed a somewhat consistent genome size range within ribotypes that clustered into two main groups with no significant intra-variability (Mann-Whitney pairwise tests; *p* < 0.01): the group made of RIBs 2, 5 and 6 displayed larger estimated genome size values than the group composed of RIBs 1, 3, 4, and 7. Such a genome size disparity likely prevents any sexual reproduction between these two groups. We additionally estimated the number of ribosomal operons per genome ranged between 58 (strain A151 belonging to RIB4) and 270 (strain A147 belonging to RIB2), with no correlation between the number of operons and the genome size (R = 0.22; *p* = 0.71) (Fig. 2B). Using the DNA-seq reads acquired for 67 individuals (17 of which were environmental “single-cells), we observed that strains in a given ribotype (Fig. 1A) are also grouped together in the *k*-mer analysis (>90%; Fig. 1E; Table S2). The results of the *k*-mer analysis suggest a low gene flow, if any, between ribotypes. Results from SSU phylogeny, CBCs, and *k*-mer analysis are consistent with placing each ribotype into a separate cryptic species, awaiting formal description.

### Correlation between “molecular” and “phenotypic” species boundaries in *Amoebophrya*

We explored whether the eight ribotypes displayed distinguishable phenotypes. Flow cytometer data showed a significant correlation between side scatter (SSC) and the forward scatter (FSC) parameters (R = 0.81; *p* < 0.01) as well as green autofluorescence (R = 0.71 and 0.94, for SSC and FSC respectively; *p* < 0.01). We frequently observed different populations of dinospores within a strain illustrated by distinct flow cytometry signatures, suggesting that dinospores could still be engaged in cell division during sporulation, as previously reported for syndinids^16,60^. FSC, SSC, and green autofluorescence differentiated strains belonging to the RIB2 from the rest, as their dinospores seemed to be brighter and larger when compared to other ribotypes (Mann-Whitney pairwise tests; *p* < 0.01) (Fig. 2C–E). We observed no significant differences among the other ribotypes for these three parameters. The separation of RIB2 (MALV-II clade 4) from the other ribotypes suggests that flow cytometry signatures can be useful for discriminating strains belonging to different higher taxonomic levels, such as various MALV-II clades as previously proposed^11^.

To explore the host range of *Amoebophrya* ribotypes, we made a strong effort to isolate the parasites and their hosts that co-occurred in the same or similar environments and were isolated during the same period of the year. As a result, representatives of the three most abundant phototrophic dinoflagellate genera (53 local strains distributed in 9 species/genetic clades) have been isolated and cross-infected in the laboratory with 36 strains representing all *Amoebophrya* ribotypes recognized in this study (excepting RIB 8, for which no strain is available) (Fig. 2F). No *Amoebophrya* strains infected the toxic dinoflagellate *Alexandrium minutum*, but all could infect all strains of *Scrippsiella acuminata* STR1. Ribotypes 1, 3, 6 and 7 only infected this species, while others infected several species in the same *Scrippsiella* genus (RIB5) or even another genus (RIB2 and RIB4 infected both *Scrippsiella* and *Heterocapsa*; Fig. 2F). We found that the capacity to infect more than one host species correlated with ribotype boundaries, where strains belonging to the same ribotype displayed similar host ranges (Fig. 2F). The overall consistency in the host spectrum observed within the different ribotypes might suggest a genetic determinism underlying host specialization. The host spectrum is often considered as more permissive in culture experiments compared to the natural environment^61^, while higher genomic diversity exists and potentially extends or reduces the host range from that observed in the laboratory. Based on the microscopic examination of the environmental single-cells at the time of their isolation, we determined that RIBs 2, 4, 5 and 8 infected both scrippsielloids and *H. triquetra*, allowing us to extend the host range determined with the cross-infection experiments (Table S1). Interestingly, RIB 3 and 8, which are closely related ribotypes based on ribosomal phylogenies but considered as different cryptic species based on CBCs and *k*-mer analysis, also differed by their host range (i.e., RIB3 infected only *S. acuminata* in the cross-infection experiments, whereas RIB8 infected both scrippsielloids and *H. triquetra* based on microscopic analysis of environmental single-cells).

We performed a principal coordinate analysis (PCoA) to assess the relative importance of the phenotypic characters and the host range in discriminating RIBs 1–7 (RIB8 was not included because no strain is available for this ribotype) (Fig. 2G). The *envfit* test indicated that the number of hosts and the genome size were the main features explaining the phenotypic discrimination of the strains (R^2^ = 0.97 and 0.96, respectively; *p* < 0.001). When used in combination, the phenotypic characters and host range allowed for the discrimination of only two ribotypes: strains of RIB4 separated from the other ribotypes based upon the highest number of potential hosts and small genome size, whereas strains of RIB6 infected only one host and had a larger genome. Overall, our results suggest that the phenotypic characters analyzed here are not sufficient to distinguish all of the *Amoebophrya* ribotypes recognized in this study, which should be considered as cryptic species.

### Application of the new species boundaries to environmental data

**Figure 3:**
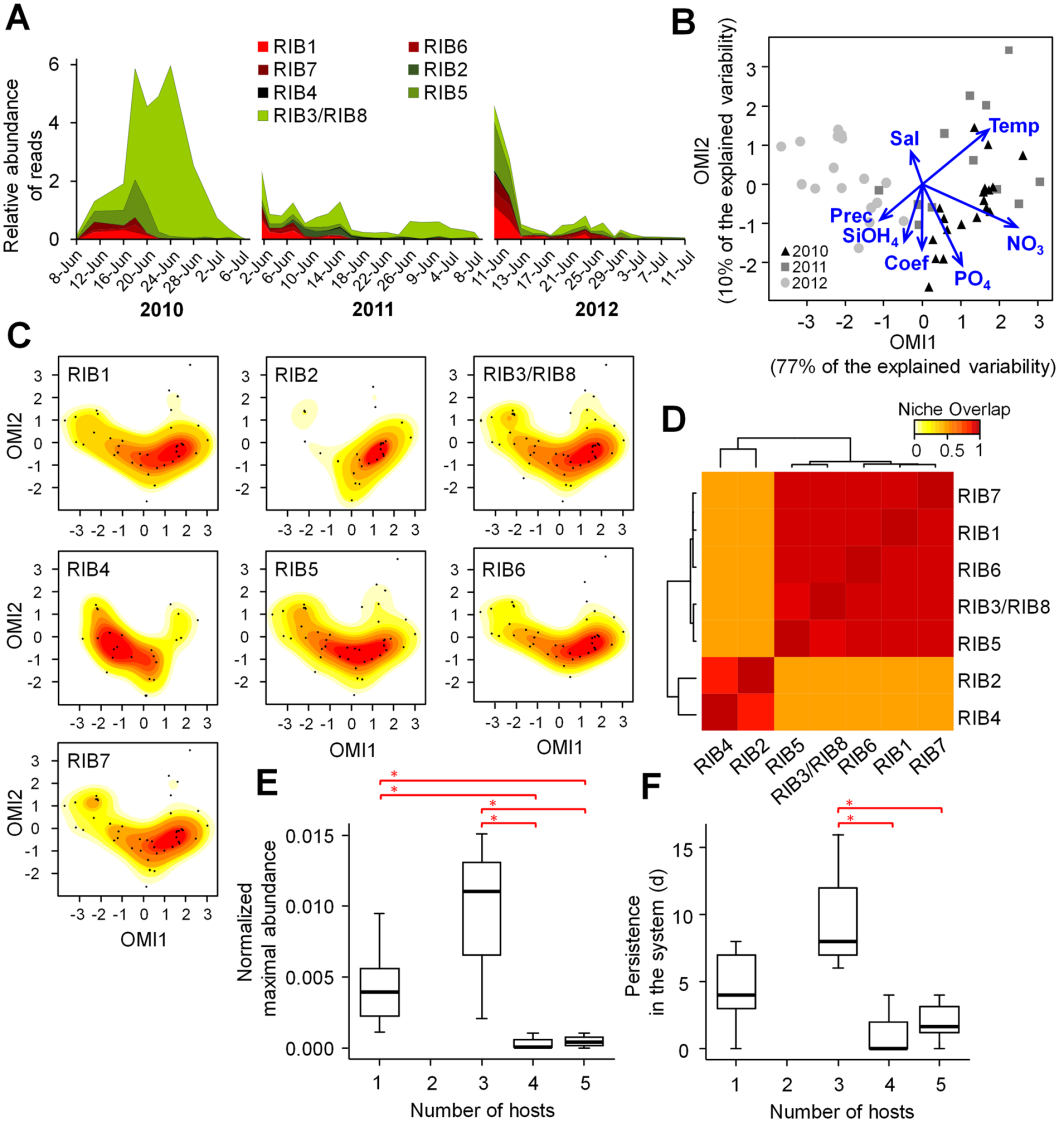
Environmental monitoring of the eight ribotypes in the Penzé estuary during a three-year survey of *Alexandrium minutum* blooms. (**A**) Relative abundance (in % of total reads) of *Amoebophrya* ribotypes in the Penzé Estuary (late spring-early summer of 2010, 2011, and 2012) based on the V4 SSU rDNA metabarcoding analysis. RIBs 3 and 8 were jointly quantified as they could not be differentiated using this marker. (**B**) Ordination diagram originated from the outlying mean index (OMI) analysis showing the distribution of the samples from the three years in the environmental space determined by the abiotic descriptors (blue vectors): temperature (Temp), salinity (Sal), precipitation (Prec), tide coefficient (Coef), and nutrients (NO_3_, PO_4_, SiOH_4_). (**C**) Distribution of the Kernel densities of the different ribotypes in the OMI multivariate space. The color gradient from yellow to red represents the density (from low to high, respectively), whereas the black dots correspond to the environmental samples shown in (**B**). (**D**) Heatmap showing similarities between ribotypes based on the pairwise *D* metric (i.e., niche overlap) calculated using the Kernel densities showed in (**C**). (**E,F**) Boxplots showing the relationship between the host range (maximal number of hosts infected by each ribotype as detected in the cross-infection experiments) and the field population fitness, defined by the normalized maximal abundance of ribotypes (**E**) and their permanence in days in the ecosystem (**F**). Horizontal lines indicate the median for the different descriptors. The red brackets indicate the significant differences between clusters pointed out by the post-hoc Dunn’s test (*p < 0.05).

As a case study, we applied the newly defined *Amoebophrya* cryptic species boundaries to a metabarcoding survey performed during toxic blooms of *A. minutum* in the Penzé Estuary over three consecutive years (2010–2012). Using a 100% threshold SSU rDNA sequence similarity (i.e., unique sequences), we identified all ribotypes except for RIBs 3 and 8 that cannot be differentiated using the V4 region (referred to as RIB3/8 hereafter). We found that all *Amoebophrya* ribotypes coexisted in the Penzé Estuary during most of the survey, but with contrasting patterns among the different years (Fig. 3A). While the proportion of *Amoebophrya*-like reads did not exceed 6% of the total reads for any given ribotype, ribotypes RIB3/8 and RIB5 were the most ubiquitous during the survey. The niche analysis based on the outlying mean index (OMI) pointed out a substantial interannual variability (Fig. 3B) mainly correlated to NO_3_ concentration and temperature levels (*envfit* test; R^2^ = 0.92 and 0.63, respectively; *p* < 0.05), both showing higher values in 2010 and 2011 than in 2012. Kernel density plots on the first two OMI axes (Fig. 3C) indicated that most ribotypes showed similar realized niches during the entire sampling period. Exceptions to this pattern were, however, observed for RIB2 and RIB4, whose occurrences were more restricted to 2010 and 2011 for RIB2 and to 2012 for RIB4. These differences were highlighted by the heatmap analysis based on the *D* metric (i.e., niche overlap) calculated using the Kernel densities (Fig. 3D), indicating a clear separation of RIBs 2 and 4 from the other ribotypes. The heatmap that took into consideration the niche overlap between *Amoebophrya* ribotypes and other dinoflagellates detected in the metabarcoding dataset (i.e., potential hosts) further indicated that RIBs 2 and 4 co-occurred with different dinoflagellate assemblages when compared to the other ribotypes (Fig. 3D). By contrast, RIBs 1 and 3–8 were in sympatry, i.e. shared the same environment and potentially the same hosts during the same period of the year. In other words, these cryptic species naturally co-occur in the Penzé estuary and potentially compete for the same resources, as cross-infection experiments indicate that they can infect the same host species.

Finally, we investigated whether the host spectrum of each ribotype (based on the maximal number of hosts detected in the cross-infection experiments) was related to its population fitness, taking into account the normalized relative abundance of reads based on the average number of operons in each ribotype. The Kruskal-Wallis test showed significant differences in the maximal normalized abundances and persistence in the environment of the *Amoebophrya* ribotypes with respect to the number of hosts that they infect (*p* < 0.05). The post-hoc Dunn’s test indicated that although no difference was observed between ribotypes with 1 and 3 hosts, with respect to the two fitness indicators (*p* > 0.05), they both showed higher maximal normalized abundances when compared with ribotypes with 4–5 hosts (*p* < 0.05; Fig. 3E). However, only ribotypes with 3 hosts persisted in the system longer when compared with ribotypes with high 4–5 hosts (*p* < 0.05) (Fig. 3F). Although these outcomes need to be interpreted with care due to the low sampling size (N = 18), they suggest a putative ecological disadvantage for *Amoebophrya* infecting an excessive number of hosts.

## Conclusions

Here, we provide molecular evidence for the presence of at least eight *Amoebophrya* ribotypes in the Penzé and Rance Estuaries, with genome *k*-mer comparisons and CBCs supporting their classification into individual cryptic *Amoebophrya* species. Our results indicate that the ITS2 region of the ribosomal operon is a better proxy than phenotypic characters for species delineation in the *A. ceratii* species complex and that nucleotide differences in the V4 SSU rDNA gene sequence might not be enough to delineate putative cryptic species. These results advocate for the use of unique sequences (i.e., 100% threshold of sequences similarity) rather than grouping them into OTUs during barcoding studies when using this genetic marker. Considering the diversity of MALV-II lineage in marine waters, a full reassessment of their taxonomy is needed to understand their biogeography and ecology. Applying this novel species definition over a three-year monitoring survey in the Penzé Estuary, we observed that most of these cryptic species co-occurred during dinoflagellate blooms, likely competing for similar ecological niches and host resources. We also reported an inverse pattern between population fitness and host range, where the maximal fitness values were observed for the *Amoebophrya* ribotypes having low to intermediate number of hosts, highlighting a higher cost for infecting a broader host range.

## Supplementary information

Supplementary Information.

## Data Availability

Raw data are available upon request or using the following link: http://application.sb-roscoff.fr/project/hapar. All strains have been deposited at the Roscoff Culture Collection (RCC, http://roscoff-culture-collection.org/).
