# Supplementary material for: Cryptic species in the parasitic Amoebophrya species complex revealed by a polyphasic approach
Source: Sci Rep. 2020 Feb 13;10:2531. doi: 10.1038/s41598-020-59524-z (PMC7018713; doi:10.1038/s41598-020-59524-z)
Supplement: Supplementary file 1 — Supplementary Information. [file 41598_2020_59524_MOESM1_ESM.docx]

**Cryptic species in the parasitic *Amoebophrya* species complex revealed by a polyphasic approach.**

Ruibo Cai^a^, Ehsan Kayal^b^, Catharina Alves-de-Souza^c^, Estelle Bigeard^a^, Erwan Corre^b^, Christian Jeanthon^a^, Dominique Marie^a^, Betina M. Porcel^d^, Raffaele Siano^e^, Jeremy Szymczak^a^, Matthias Wolf^f^, Laure Guillou^a,1^

**^a^**Sorbonne Université, CNRS, UMR7144 Adaptation et Diversité en Milieu Marin, Ecology of Marine Plankton (ECOMAP), Station Biologique de Roscoff SBR, 29680 Roscoff, France

**^b^**Sorbonne Université, CNRS, FR2424 ABIMS, Station Biologique de Roscoff SBR, 29680 Roscoff, France

**^c^**Algal Resources Collection, MARBIONC, Center for Marine Sciences, University of North Carolina Wilmington, 5600 Marvin K. Moss Lane, Wilmington, NC 28409, US

**^d^**Génomique Métabolique, Genoscope, Institut François Jacob, CEA, CNRS, Univ. Evry, Université Paris-Saclay, 91057 Evry, France

**^e^**Ifremer-Centre de Bretagne, Département/Unité/Laboratoire ODE/DYNECO/Pelagos, Z.I. Technopôle Brest-Iroise, Pointe du Diable BP70, 29280 Plouzané, France

**^f^**Department of Bioinformatics, Biocenter, University of Würzburg, Am Hubland, 97074 Würzburg, Germany

^1^To whom correspondence should be addressed. Email:lguillou@sb-roscoff.fr. Laure Guillou, Sorbonne Université, CNRS, UMR7144 Adaptation et Diversité en Milieu Marin, Ecology of Marine Plankton (ECOMAP), Station Biologique de Roscoff SBR, 29680 Roscoff, France. +33667972473.

**SUPPLEMENTARY INFORMATION**

This document contains supplementary methods of culturing effort and genome analysis.

**Supplementary methods**

**1. Culturing**

Host and parasite strains were grown in F/2 medium (Marine Water Enrichment Solution, Sigma), using filtered (0.22 µm) and autoclaved natural seawater from the Penzé Estuary (27 practical salinity units) after it was stored in the dark for over 3 months. The medium was supplemented with 5% (v/v) soil extract followed by a final filtration (0.22 µm) under sterile conditions. Cultures were grown at 21°C in vented flasks under a 12h:12h light:dark photoperiod at 100 µEinstein m^2^ s^−1^. Parasitic strains were maintained by transferring infected hosts into healthy host cultures every 3-7 days in 15 ml culture tubes and using a 1:10 parasite:host ratio.

**Isolation of dinoflagellates hosts**

Cultures of the putative dinoflagellates hosts were isolated by micropipetting during the entire monitoring period and later used to maintain the *Amoebophrya* strains as well as to assess their host range.

**Isolation of *Amoebophrya* strains**

Wells of 24-well plates were incubated with 1 ml of healthy host strains (or a mix of strains) and then supplemented with either 1) 1 ml of field sample filtered through polycarbonate filters (3-5 µm pore size) (fraction presumably containing dinospores; i.e., their free-living, infective stage), or 2) one infected host cell isolated by micropipetting (Box 1). Similar percentages of infection success were obtained using both strategies (8-10 %), with more chance of success observed when a mix of host species was used.

**Box 1: Percentage of successful infections obtained with the two isolation strategies (i.e., direct incubation of field filtered seawater or isolated single infected host cell).**

| Host species | Number of incubations | Number of starting infections | Success rate (%) |
| --- | --- | --- | --- |
| *Alexandrium minutum* | 438 | 0 | 0 |
| *Gymnodinium* sp*.* | 7 | 0 | 0 |
| *Heterocapsa rotundata* | 12 | 0 | 0 |
| *Heterocapsa triquetra* | 194 | 5 | 2.58 |
| *Prorocentrum micans* | 9 | 0 | 0 |
| *Scrippsiella donghaienis* | 166 | 17 | 10.24 |
| *Scrippsiella acuminata* STR1 | 180 | 50 | 27.78 |
| *Scrippsiella* sp. 1 | 2 | 0 | 0 |
| Mix of species | 60 | 25 | 41.67 |
| Total | **1068** | **97** | **9.08** |
|  |  |  |  |
| Isolation strategy |  |  |  |
| by incubation of field water | 976 | 83 | 8.50 |
| by isolating one infected host | 195 | 21 | 10.77 |

Plates were checked for *Amoebophrya*-like parasites through their natural green autofluorescence using an epifluorescence microscope (BX51, Olympus) equipped with the U-MWB2 cube [450- to 480-nm excitation, 500-nm emission (Coats and Bockstahler 1994. J Eukaryot Microbiol. 1994;41(6):586–93.)]. Overall, infections were observed after 3-7 days in 9.08 % of the cases (deduced from the 1,068 incubations processed in 2010 and 2011), with success rates that depended on the host species: no infection in *A. minutum*, 2.6% in *H. triquetra*, 10.2% in *S. donghaienis*, 27.8% in *S. acuminata* STR1 type 1, and 41.7% using mixes of species. For the establishment of the strains, a single infected host cell was isolated from those incubations by micropipeting (only one kept per well), washed three times and newly transferred into healthy cultures of the host where infections were first observed. The clonality of the strains was ensured by repeating this step 2-5 times.

**2. Rationale for not using ultrastructure of *Amoebophrya***

Morphology and SEM have long been used for taxonomy purposes. For instance, in its initial description, Cachon [1964, Ann des Sci Nat Zool Paris. (12ème série): 1-158] defined species boundaries within Amoebophryidae based on the specific configuration of the cytopharynx, a structure responsible for the transit of particles from the host to the parasite during the internal developmental stages (i.e., trophont). However, the morphology of Amoebophrya intracellular stages changes rapidly (in a matter of hours), from one aflagellate cell to thousands of flagellate inoculums. The sporulation starts early during the parasitic development, and there is no clear separation between the trophont and the sporont stages. This is clearly a problem when the same stage should be compared over different parasitic strains, and definitely challenging to be applied for the comparison of hundreds of strains. We also observed that the infection mode of Amoebophrya depends on the nature and the physiology of its host. For instance, infections can be cytoplasmic or nuclear, depending on the host. As ultrastructure of intracellular stages of symbionts, in general, is highly dependent upon the physiology of the host and the number of co-infections (Figueroa et al. 2008, Protist 159: 563-578), free-living stages should be preferred for taxonomic purposes of endoparasites (see Lepelletier et al. 2014, Protist 165: 230-244.). Amoebophrya spp. dinospores are, however, small (less than 3-5 µm in diameter), short-living (2-3 days), and submitted to rapid size variation as additional cell divisions happen just after their release (this study). Their size, infectivity, motility and viability dramatically change over a few hours (Coats and Park 2002, Aquat Microb Ecol. 528: 520-528). Consequently, there is no Amoebophrya life stage showing stable morphological characters allowing for reliable comparison between strains by electron microscopy (such as the resting stages commonly used for solving the taxonomy of dinoflagellates). This is the reason why we based our phenotypic analysis on flow cytometry, a reliable technique that additionally allows for the rapid analysis of large populations of living cells rather than time-consuming techniques such as electron microscopy that can only focus on few cells at a time.

**3. Genome analysis**

**Sequencing steps**

Total DNA was quantified on a Qubit Fluorometer whereas DNA quality was checked by electrophoresis on a 0.7% agarose gel. We prepared overlapping paired-end libraries from 250 ng of the total DNA using a semi-automated protocol. Briefly, DNA was sheared on a Covaris E210 ultrasonicator (Covaris, Inc., Woburn, Massachusetts, USA) in order to generate fragments of 150-400 bp in size. End repair, A-tailing and ligation with Illumina compatible adaptors (Bio Scientific Austin, Texas, USA) were performed using the SPRIWorks Library Preparation System and an SPRI TE instrument (Beckmann Coulter, Danvers, Massachusetts, USA) according to the manufacturer’s protocol. The 200-400 bp size fragments were amplified by 12 cycles of PCR with the Pfx Platinum Taq polymerase (ThermoFisher, Waltham, Massachusetts, USA) and Illumina adapter-specific primers. Amplified library fragments were size selected on a 3% agarose gel around 300 bp and purified. We prepared a mate-pair (MP) library for A25 using 10 µg of fragmented DNA according to the Illumina protocol (Illumina Mate Pair library kit, Illumina, San Diego, CA). For strain A120, the MP library was prepared with the Nextera Mate Pair Sample Preparation Kit (Illumina) using 4 µg of fragmented DNA.

We evaluated the size of all Illumina libraries on an Agilent 2100 Bioanalyzer (Agilent Technologies, Palo Alto, CA, USA) machine and quantified them by qPCR with the KAPA Library Quantification Kit (KapaBiosystems Inc., Woburn, MA, USA) on a MxPro instrument (Agilent Technologies). Libraries were then sequenced using the 101 bp paired-end reads chemistry on a HiSeq2000 Illumina sequencer. Few more individuals have been sequenced on an Illumina HiSeq XTEN or BGISEQ-500 platform in BGI (Box 2). After filtering off duplicated, low quality reads and reads with adaptor sequences, 3 - 6 Gb (~15-30 X genome sequencing depth high-quality clean reads were retained for each sample.

**Assembling of genomes**


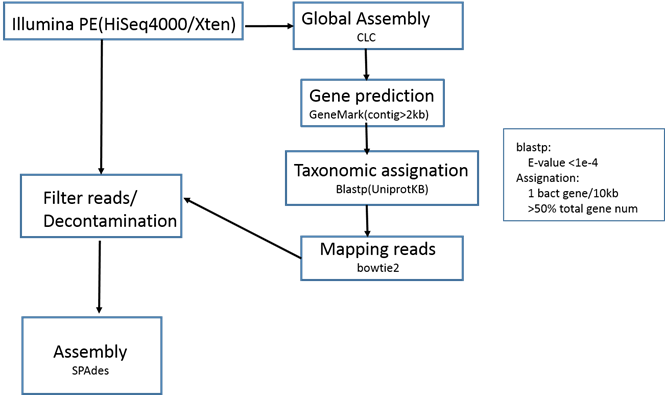
A first assembly was processed using CLC assembler, clc_mapper, with the options (-p fb ss 200 800 –q). The bioinformatics pipeline was then customized to remove bacterial contamination in the chart flow below:

We additionally confirmed the identity of each individual by comparing the partial ribosomal operon (SSU rDNA, ITS1, 5.8S, ITS2) extracted from contigs from the one obtained by PCR.

**Figure S1: Phylogeny of Syndiniales Group II (MALV-II) based upon the V4 region of the SSU rDNA gene.** Collapsed PhyML phylogenetic tree of 1,550 sequences 511 bp in length of the SSU rDNA corresponding to the V4 region using the GTR + G model. All selected strains from this study (in blue) belong to the MALVII (Amoebophryidae) clades 2 and 4 nomenclature following the one from Guillou et al. (2008). Terminal values represent the number of sequences per clade. Bootstraps values based on 100 replicates of terminal nodes are shown for the MALVII clades 2 and 4.


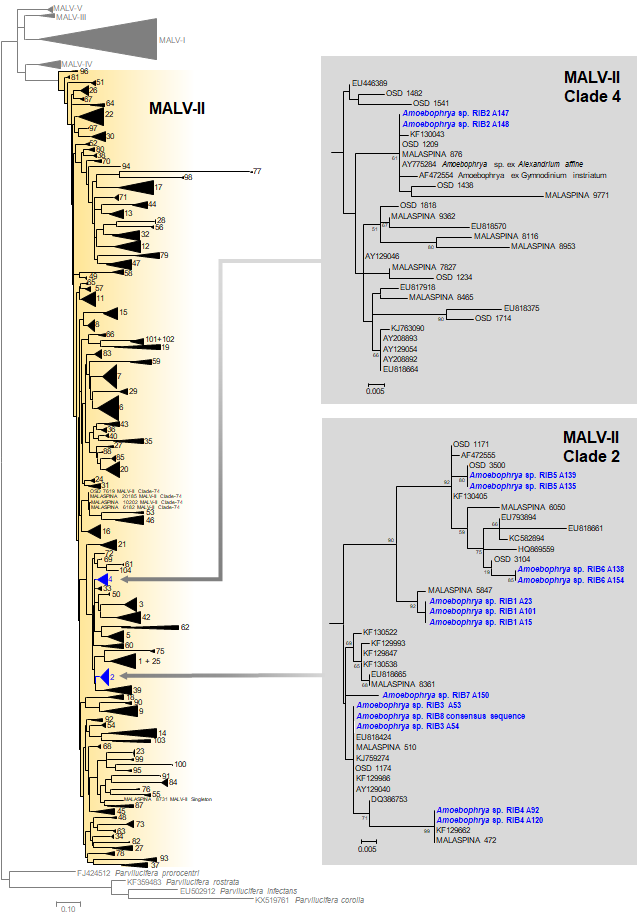


**Figure S2: Phylogeny of the *Scrippsiella* spp. strains used in this study based on the D1-D2 domains of the LSU rDNA gene.** PhyML phylogeny based upon analysis of sequences 705 bp in length of the D1-D2 region of the LSU rDNA gene using the GTR+G model. Bootstrap values (> 70%) based on 100 replicates for the main clades are shown. We based our nomenclature of scrippsielloids upon a phylogeny using the D1 and D2 domains of the LSU rDNA genes, after taxonomy of Luo et al. 2016. However, the taxonomy of scrippsielloids is still under construction by experts. The most common species of *Scrippsiella*, i.e. *S. trochoidea* (F.Stein) A.R.Loebl., comprises three genetically diverse clades, designated as STR1, STR2 and STR3 (Montresor et al. 2003, Phycologia. 42:56–70, Gottschling et al. 2005, Eur J Phycol. 2005;40(2):207–20). These genetic clusters should be considered as distinct species. Recently, strains of *S. trochoidea* from the type locality proved to be in STR2, thus STR1 and STR3 might not be true *S. trochoidea* at all (Zinssmeister et al. 2012, J Phycol. 48(5):1107–18, Soehner et al. 2012, Organisms Diversity & Evol. 12:339–48). The true *S. trochoidea* (STR2) is now considered a heterotypic synonym of *S. acuminata* (Ehrenb.) Kretschmann, Elbr., Zinssmeister, S. Soehner, Kirsch, Kusber & Gottschling (Kretschmann et al. 2015, Phytotaxa. 17(3):239–56), a change that we take into account here, in complementary to the assignation to the genetic clades, awaiting formal description.


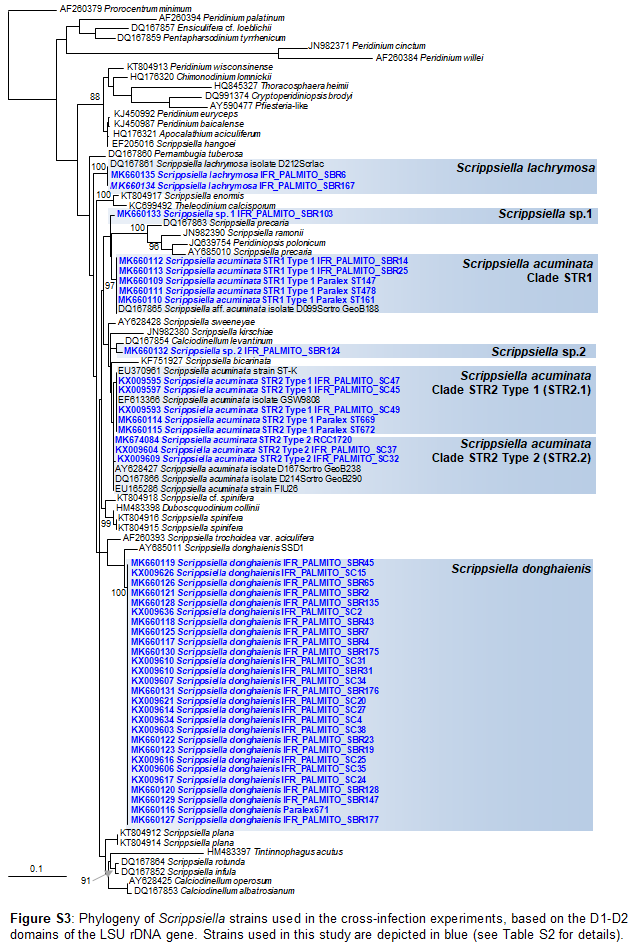


**Table S1: List of strains and single-cells.** This table includes species identity of individuals, their identifiers, RCC number (Roscoff Culture Collection, http://roscoff-culture-collection.org/), GenBank accession numbers for marker genes, host for parasitic strains, geographical origin, and date of isolation. The three last columns indicate in which kind of analysis the different strains were used.

| Species | Mode | ID_strain/SC | | Host_during isolation | Host_present | Roscoff Culture Collection (RCC) | GenBank acc. Number | Origin | Date of isolation (datation of sediment) | Use for ribotyping | Use for cross-infection | Use for whole genome sequencing |
| --- | --- | --- | --- | --- | --- | --- | --- | --- | --- | --- | --- | --- |
| Amoebophrya RIB1 | Strain | A1 | | ST147 | ST161 | RCC5984 | XXXXXXXXX (18S, ITS1-5.8S-ITS2) | Penzé estuary | 23/06/2007 | **X** | **-** | **X** |
| Amoebophrya RIB6 | Strain | A100 | | ST161 | ST161 | RCC5997 | XXXXXXXXX (18S, ITS1-5.8S-ITS2) | Penzé estuary | 01/06/2011 | **X** | **-** | **X** |
| Amoebophrya RIB1 | Strain | A101 | | ST161 | ST161 | RCC4387 | XXXXXXXXX (ITS1, 5.8S, ITS2) | Penzé estuary | 01/06/2011 | **X** | **X** | **-** |
| Amoebophrya RIB6 | Strain | A102 | | ST147 | ST161 | RCC6079 | XXXXXXXXX (ITS1, 5.8S, ITS2) | Penzé estuary | 01/06/2011 | **X** | **-** | **-** |
| Amoebophrya RIB6 | Strain | A103 | | ST161 | ST161 | RCC5998 | XXXXXXXXX (18S, ITS1-5.8S-ITS2) | Penzé estuary | 01/06/2011 | **X** | **-** | **X** |
| Amoebophrya RIB1 | Strain | A104 | | ST147 | ST147 | LOST | XXXXXXXXX (ITS1, 5.8S, ITS2) | Penzé estuary | 03/06/2011 | **X** | **-** | **-** |
| Amoebophrya RIB1 | Strain | A105 | | ST147 | ST161 | RCC4388 | XXXXXXXXX (ITS1, 5.8S, ITS2) | Penzé estuary | 03/06/2011 | **X** | **X** | **-** |
| Amoebophrya RIB6 | Strain | A106 | | ST147 | ST161 | RCC5999 | XXXXXXXXX (18S, ITS1-5.8S-ITS2) | Penzé estuary | 03/06/2011 | **X** | **-** | **X** |
| Amoebophrya RIB6 | Strain | A107 | | ST161 | ST161 | RCC6000 | XXXXXXXXX (18S, ITS1-5.8S-ITS2) | Penzé estuary | 03/06/2011 | **X** | **-** | **X** |
| Amoebophrya RIB3 | Strain | A108 | | ST147 | ST147 | LOST | XXXXXXXXX (ITS1, 5.8S, ITS2) | Penzé estuary | 07/06/2011 | **X** | **-** | **-** |
| Amoebophrya RIB6 | Strain | A109 | | ST161 | ST161 | RCC6080 | XXXXXXXXX (ITS1, 5.8S, ITS2) | Penzé estuary | 07/06/2011 | **X** | **-** | **-** |
| Amoebophrya RIB1 | Strain | A110 | | ST147 | ST161 | RCC6001 | XXXXXXXXX (18S, ITS1-5.8S-ITS2) | Penzé estuary | 07/06/2011 | **X** | **-** | **X** |
| Amoebophrya RIB6 | Strain | A111 | | ST161 | ST161 | RCC6002 | XXXXXXXXX (18S, ITS1-5.8S-ITS2) | Penzé estuary | 08/06/2011 | **X** | **-** | **X** |
| Amoebophrya RIB6 | Strain | A112 | | ST147 | ST161 | RCC6081 | XXXXXXXXX (ITS1, 5.8S, ITS2) | Penzé estuary | 08/06/2011 | **X** | **-** | **-** |
| Amoebophrya RIB6 | Strain | A114 | | ST147 | ST161 | RCC6003 | XXXXXXXXX (18S, ITS1-5.8S-ITS2) | Penzé estuary | 13/06/2011 | **X** | **-** | **X** |
| Amoebophrya RIB6 | Strain | A116 | | ST147 | ST161 | RCC4399 | XXXXXXXXX (ITS1, 5.8S, ITS2) | Penzé estuary | 13/06/2011 | **X** | **X** | **-** |
| Amoebophrya RIB6 | Strain | A117 | | ST147 | ST161 | RCC6004 | XXXXXXXXX (18S, ITS1-5.8S-ITS2) | Penzé estuary | 13/06/2011 | **X** | **-** | **X** |
| Amoebophrya RIB1 | Strain | A12 | | ST147 | ST161 | RCC4382 | XXXXXXXXX (ITS1, 5.8S, ITS2) | Penzé estuary | 23/06/2007 | **X** | **X** | **-** |
| Amoebophrya RIB4 | Strain | A120 | | HT150 | HT150 | RCC4398 | XXXXXXXXX (18S, ITS1, 5.8S, ITS2) from Matthieu | Penzé estuary | 13/06/2011 | **X** | **X** | **-** |
| Amoebophrya RIB6 | Strain | A121 | | ST161 | ST161 | RCC4409 | XXXXXXXXX (ITS1, 5.8S, ITS2) | Penzé estuary | 14/06/2011 | **X** | **X** | **-** |
| Species | Mode | ID_strain/SC | | Host_during isolation | Host_present | Roscoff Culture Collection (RCC) | GenBank acc. Number | Origin | Date of isolation (datation of sediment) | **Use for r**ibotyping | **Use for c**ross-infection | **Use for w**hole genome **sequencing** |
| Amoebophrya RIB1 | Strain | A123 | | ST147 | ST161 | RCC6082 | XXXXXXXXX (ITS1, 5.8S, ITS2) | Penzé estuary | 14/06/2011 | **X** | **-** | **-** |
| Amoebophrya RIB1 | Strain | A124 | | ST161 | ST161 | RCC4389 | XXXXXXXXX (ITS1, 5.8S, ITS2) | Penzé estuary | 14/06/2011 | **X** | **X** | **-** |
| Amoebophrya RIB6 | Strain | A126 | | ST161 | ST161 | RCC4410 | XXXXXXXXX (18S, ITS1-5.8S-ITS2) | Penzé estuary | 14/06/2011 | **X** | **X** | **X** |
| Amoebophrya RIB6 | Strain | A127 | | ST161 | ST161 | RCC6005 | XXXXXXXXX (18S, ITS1-5.8S-ITS2) | Penzé estuary | 14/06/2011 | **X** | **-** | **X** |
| Amoebophrya RIB4 | Strain | A129 | | HT150 | HT150 | RCC6006 | XXXXXXXXX (18S, ITS1-5.8S-ITS2) | Penzé estuary | 14/06/2011 | **X** | **-** | **X** |
| Amoebophrya RIB5 | Strain | A135 | | ST147 | ST161 | RCC4402 | XXXXXXXXX (18S, ITS1, 5.8S, ITS2) | Rance estuary | 29/05/2011 | **X** | **X** | **X** |
| Amoebophrya RIB6 | Strain | A136 | | ST147 | ST161 | RCC6007 | XXXXXXXXX (18S, ITS1-5.8S-ITS2) | Rance estuary | 01/06/2011 | **X** | **-** | **X** |
| Amoebophrya RIB6 | Strain | A137 | | ST147 | ST161 | RCC4411 | XXXXXXXXX (18S, ITS1-5.8S-ITS2) | Rance estuary | 01/06/2011 | **X** | **X** | **X** |
| Amoebophrya RIB6 | Strain | A138 | | ST147 | ST161 | RCC4413 | XXXXXXXXX (18S, ITS1-5.8S-ITS2) | Rance estuary | 05/06/2011 | **X** | **X** | **X** |
| Amoebophrya RIB5 | Strain | A139 | | ST147 | ST161 | RCC4403 | XXXXXXXXX (18S, ITS1-5.8S-ITS2) | Rance estuary | 01/06/2011 | **X** | **X** | **X** |
| Amoebophrya RIB6 | Strain | A141 | | ST147 | ST161 | RCC4412 | XXXXXXXXX (18S, ITS1-5.8S-ITS2) | Rance estuary | 08/06/2011 | **X** | **X** | **X** |
| Amoebophrya RIB5 | Strain | A142 | | ST147 | ST161 | RCC4401 | XXXXXXXXX (18S, ITS1-5.8S-ITS2) | Rance estuary | 22/06/2011 | **X** | **X** | **X** |
| Amoebophrya RIB6 | Strain | A144 | | ST147 | ST161 | RCC6008 | XXXXXXXXX (18S, ITS1-5.8S-ITS2) | Rance estuary | 01/06/2011 | **X** | **-** | **X** |
| Amoebophrya RIB6 | Strain | A145 | | ST161 | ST161 | RCC4414 | XXXXXXXXX (ITS1, 5.8S, ITS2) | Rance estuary | 05/06/2011 | **X** | **X** | **-** |
| Amoebophrya RIB6 | Strain | A146 | | ST161 | ST161 | RCC6009 | XXXXXXXXX (18S, ITS1-5.8S-ITS2) | Rance estuary | 05/06/2011 | **X** | **-** | **X** |
| Amoebophrya RIB2 | Strain | A147 | | ST147 | ST161 | RCC4390 | XXXXXXXXX (18S, ITS1, 5.8S, ITS2) | Penzé estuary | 08/07/2011 | **X** | **X** | **X** |
| Amoebophrya RIB2 | Strain | A148 | | ST147 | ST161 | RCC4391 | XXXXXXXXX (ITS1, 5.8S, ITS2) | Penzé estuary | 08/07/2011 | **X** | **X** | **-** |
| Amoebophrya RIB2 | Strain | A149 | | ST147 | ST161 | RCC4392 | XXXXXXXXX (18S, ITS1, 5.8S, ITS2) | Penzé estuary | 08/07/2011 | **X** | **X** | **X** |
| Amoebophrya RIB1 | Strain | A15 | | ST147 | ST161 | RCC4381 | HQ658161 (18S), XXXXXXXXX (ITS1, 5.8S, ITS2) | Penzé estuary | 23/06/2007 | **X** | **X** | **-** |
| Amoebophrya RIB7 | Strain | A150 | | ST161 | ST161 | RCC4416 | XXXXXXXXX (18S, ITS1-5.8S-ITS2) | Penzé estuary | 08/07/2011 | **X** | **X** | **X** |
| Amoebophrya RIB4 | Strain | A151 | | HT150 | HT150 | LOST | XXXXXXXXX (18S, ITS1, 5.8S, ITS2) | Penzé estuary | 08/07/2011 | **X** | **-** | **X** |
| Amoebophrya RIB6 | Strain | A152 | | ST161 | ST161 | RCC4393 | XXXXXXXXX (18S, ITS1-5.8S-ITS2) | Penzé estuary | 08/07/2011 | **X** | **X** | **X** |
| Amoebophrya RIB1 | Strain | A153 | | ST161 | ST161 | RCC6083 | XXXXXXXXX (ITS1, 5.8S, ITS2) | Penzé estuary | 01/06/2011 | **X** | **-** | **-** |
| Amoebophrya RIB2 | Strain | A154 | | ST161 | ST161 | RCC6010 | XXXXXXXXX (18S, ITS1-5.8S-ITS2) | Penzé estuary | 08/06/2011 | **X** | **-** | **X** |
| Species | Mode | ID_strain/SC | | Host_during isolation | Host_present | Roscoff Culture Collection (RCC) | GenBank acc. Number | Origin | Date of isolation (datation of sediment) | **Use for r**ibotyping | **Use for c**ross-infection | **Use for w**hole genome **sequencing** |
| Amoebophrya RIB1 | Strain | A24 | | ST147 | ST161 | RCC5985 | XXXXXXXXX (18S, ITS1-5.8S-ITS2) | Penzé estuary | 15/06/2009 | **X** | **-** | **X** |
| Amoebophrya RIB1 | Strain | A25 | | ST147 | ST161 | RCC4383 | XXXXXXXXX (ITS1, 5.8S, ITS2) | Penzé estuary | 15/06/2009 | **X** | **X** | **-** |
| Amoebophrya RIB1 | Strain | A29 | | ST147 | ST161 | RCC5986 | XXXXXXXXX (18S, ITS1-5.8S-ITS2) | Penzé estuary | 15/06/2009 | **X** | **-** | **X** |
| Amoebophrya RIB1 | Strain | A30 | | ST147 | ST161 | RCC5987 | XXXXXXXXX (18S, ITS1-5.8S-ITS2) | Penzé estuary | 15/06/2009 | **X** | **-** | **X** |
| Amoebophrya RIB1 | Strain | A32 | | ST147 | ST161 | LOST | XXXXXXXXX (18S, ITS1, 5.8S, ITS2) | Penzé estuary | 15/06/2009 | **X** | **-** | **X** |
| Amoebophrya RIB1 | Strain | A33 | | ST147 | ST161 | RCC5988 | XXXXXXXXX (18S, ITS1-5.8S-ITS2) | Penzé estuary | 15/06/2009 | **X** | **-** | **X** |
| Amoebophrya RIB1 | Strain | A34 | | ST147 | ST161 | RCC5989 | XXXXXXXXX (18S, ITS1-5.8S-ITS2) | Penzé estuary | 15/06/2009 | **X** | **-** | **X** |
| Amoebophrya RIB4 | Strain | A37 | | ST147 | ST161 | RCC5990 | XXXXXXXXX (18S, ITS1-5.8S-ITS2) | Penzé estuary | 18/06/2009 | **X** | **-** | **X** |
| Amoebophrya RIB4 | Strain | A42 | | ST147 | ST161 | RCC4395 | XXXXXXXXX (18S, ITS1, 5.8S, ITS2) | Penzé estuary | 18/06/2009 | **X** | **X** | **X** |
| Amoebophrya RIB4 | Strain | A46 | | ST147 | HT150 | RCC5991 | XXXXXXXXX (18S, ITS1-5.8S-ITS2) | Penzé estuary | 18/06/2009 | **X** | **-** | **X** |
| Amoebophrya RIB4 | Strain | A48 | | ST147 | ST161 | RCC4396 | XXXXXXXXX (18S, ITS1, 5.8S, ITS2) | Penzé estuary | 18/06/2009 | **X** | **X** | **X** |
| Amoebophrya RIB1 | Strain | A49 | | ST147 | ST161 | RCC5992 | XXXXXXXXX (18S, ITS1-5.8S-ITS2) | Penzé estuary | 19/06/2009 | **X** | **-** | **X** |
| Amoebophrya RIB1 | Strain | A51 | | ST147 | ST161 | RCC5993 | XXXXXXXXX (18S, ITS1-5.8S-ITS2) | Penzé estuary | 19/06/2009 | **X** | **-** | **X** |
| Amoebophrya RIB1 | Strain | A52 | | ST147 | ST161 | RCC4384 | XXXXXXXXX (ITS1, 5.8S, ITS2) | Penzé estuary | 19/06/2009 | **X** | **X** | **-** |
| Amoebophrya RIB3 | Strain | A54 | | ST147 | ST161 | RCC4394 | XXXXXXXXX (18S, ITS1, 5.8S, ITS2) | Penzé estuary | 19/06/2009 | **X** | **X** | **X** |
| Amoebophrya RIB6 | Strain | A71 | | ST161 | ST161 | RCC4404 | XXXXXXXXX (18S, ITS1-5.8S-ITS2) | Penzé estuary | 11/06/2010 | **X** | **X** | **X** |
| Amoebophrya RIB6 | Strain | A72 | | ST161 | ST161 | RCC4405 | XXXXXXXXX (18S, ITS1-5.8S-ITS2) | Penzé estuary | 11/06/2010 | **X** | **X** | **X** |
| Amoebophrya RIB6 | Strain | A74 | | ST161 | ST161 | RCC4406 | XXXXXXXXX (18S, ITS1-5.8S-ITS2) | Penzé estuary | 11/06/2010 | **X** | **X** | **X** |
| Amoebophrya RIB1 | Strain | A75 | | ST161 | ST161 | RCC4385 | XXXXXXXXX (18S, ITS1, 5.8S, ITS2) | Penzé estuary | 11/06/2010 | **X** | **X** | **X** |
| Amoebophrya RIB5 | Strain | A76 | | ST161 | ST161 | RCC4400 | XXXXXXXXX (18S, ITS1, 5.8S, ITS2) | Penzé estuary | 11/06/2010 | **X** | **X** | **X** |
| Amoebophrya RIB1 | Strain | A77 | | ST161 | ST161 | RCC6084 | XXXXXXXXX (ITS1, 5.8S, ITS2) | Penzé estuary | 18/06/2010 | **X** | **-** | **-** |
| Amoebophrya RIB7 | Strain | A78 | | ST161 | ST161 | RCC4415 | XXXXXXXXX (18S,ITS1-5.8S-ITS2) | Penzé estuary | 03/07/2010 | **X** | **X** | **X** |
| Amoebophrya RIB6 | Strain | A79 | | ST161 | ST161 | RCC4407 | XXXXXXXXX (ITS1, 5.8S, ITS2) | Penzé estuary | 03/07/2010 | **X** | **X** | **-** |
| Amoebophrya RIB1 | Strain | A80 | | ST161 | ST161 | RCC4386 | XXXXXXXXX (ITS1, 5.8S, ITS2) | Penzé estuary | 03/07/2010 | **X** | **X** | **-** |
| Amoebophrya RIB4 | Strain | A91 | | HT150 | HT150 | RCC5994 | XXXXXXXXX (18S, ITS1-5.8S-ITS2) | Penzé estuary | 18/06/2009 | **X** | **-** | **X** |
| Species | Mode | ID_strain/SC | | Host_during isolation | Host_present | Roscoff Culture Collection (RCC) | GenBank acc. Number | Origin | Date of isolation (datation of sediment) | **Use for r**ibotyping | **Use for c**ross-infection | **Use for w**hole genome **sequencing** |
| Amoebophrya RIB4 | Strain | A92 | | HT150 | HT150 | RCC6085 | XXXXXXXXX (ITS1, 5.8S, ITS2) | Penzé estuary | 18/06/2009 | **X** | **-** | **-** |
| Amoebophrya RIB4 | Strain | A93 | | HT150 | HT150 | RCC4397 | XXXXXXXXX (ITS1, 5.8S, ITS2) | Penzé estuary | 18/06/2009 | **X** | **X** | **-** |
| Amoebophrya RIB6 | Strain | A95 | | ST147 | ST161 | RCC6096 | XXXXXXXXX (ITS1, 5.8S, ITS2) | Penzé estuary | 01/06/2011 | **X** | **-** | **-** |
| Amoebophrya RIB1 | Strain | A96 | | ST147 | ST161 | RCC6087 | XXXXXXXXX (ITS1, 5.8S, ITS2) | Penzé estuary | 01/06/2011 | **X** | **-** | **-** |
| Amoebophrya RIB6 | Strain | A97 | | ST147 | ST161 | RCC5995 | XXXXXXXXX (18S, ITS1-5.8S-ITS2) | Penzé estuary | 01/06/2011 | **X** | **-** | **X** |
| Amoebophrya RIB6 | Strain | A98 | | ST161 | ST161 | RCC4408 | XXXXXXXXX (18S, ITS1-5.8S-ITS2) | Penzé estuary | 01/06/2011 | **X** | **X** | **X** |
| Amoebophrya RIB6 | Strain | A99 | | ST161 | ST161 | RCC6088 | XXXXXXXXX (18S, ITS1-5.8S-ITS2) | Penzé estuary | 01/06/2011 | **X** | **-** | **X** |
| *Scrippsiella acuminata* STR1 | Strain | Paralex 147 | | NA | NA | RCC1627 | MK660109 (LSU) | Penzé estuary | 2005 | **X** | **X** | **-** |
| *Heterocapsa triquetra* | Strain | Paralex 150 | | NA | NA | RCC3596 | MK660139 (LSU) | Penzé estuary | 06/07/2007 | **X** | **X** | **-** |
| *Scrippsiella acuminata* STR1 | Strain | Paralex 161 | | NA | NA | RCC6094 | MK660110 (LSU) | Penzé estuary | 2005 | **X** | **X** | **-** |
| *Alexandrium minutum* | Strain | Paralex 176 | | NA | NA | RCC3018 | MK660136 (LSU) | Morlaix Bay | 1989 | **X** | **X** | **-** |
| *Alexandrium minutum* | Strain | Paralex 331 | | NA | NA | RCC3145 | MK660137 (LSU) | Penzé estuary | 02/06/2010 | **X** | **X** | **-** |
| *Heterocapsa triquetra* | Strain | Paralex 36 | | NA | NA | LOST | MK660140 (LSU) | Penzé estuary | 28/06/2007 | **X** | **X** | **-** |
| *Scrippsiella acuminata* STR1 | Strain | Paralex 478 | | NA | NA | RCC3048 | MK660111 (LSU) | Penzé estuary | 22/06/2010 | **X** | **X** | **-** |
| *Heterocapsa triquetra* | Strain | Paralex 668 | | NA | NA | RCC3044 | MK660141 (LSU) | Penzé estuary | 11/07/2011 | **X** | **X** | **-** |
| *Scrippsiella acuminata* STR2 Type 1 | Strain | Paralex 669 | | NA | NA | RCC3049 | MK660114 (LSU) | Penzé estuary | 11/07/2011 | **X** | **X** | **-** |
| *Heterocapsa triquetra* | Strain | Paralex 670 | | NA | NA | RCC3043 | MK660142 (LSU) | Penzé estuary | 11/07/2011 | **X** | **X** | **-** |
| *Scrippsiella donghaienis* | Strain | Paralex 671 | | NA | NA | RCC3047 | MK660116 (LSU) | Penzé estuary | 11/07/2011 | **X** | **X** | **-** |
| *Scrippsiella acuminata* STR2 Type 1 | Strain | Paralex 672 | | NA | NA | LOST | MK660115 (LSU) | Penzé estuary | 11/07/2011 | **X** | **X** | **-** |
| *Heterocapsa triquetra* | Strain | Paralex 694 | | NA | NA | LOST | MK660143 (LSU) | Penzé estuary | 15/06/2011 | **X** | **X** | **-** |
| *Heterocapsa triquetra* | Strain | Paralex 836 | | NA | NA | LOST | MK660144 (LSU) | Penzé estuary | 11/06/2011 | **X** | **X** | **-** |
| Species | Mode | ID_strain/SC | | Host_during isolation | Host_present | Roscoff Culture Collection (RCC) | GenBank acc. Number | Origin | Date of isolation (datation of sediment) | **Use for r**ibotyping | **Use for c**ross-infection | **Use for w**hole genome **sequencing** |
| *Alexandrium minutum* | Strain | Paralex 873 | | NA | NA | RCC3278 | MK660138 (LSU) | Rance estuary | 05/06/2011 | **X** | **X** | **-** |
| *Scrippsiella sp.2* | Strain | IFR_PALMITO_SBR103 | | NA | NA | RCC6113 | MK660133 (LSU) | Penzé estuary | 08/04/2014 (2000 +/- 4.1) | **X** | **X** | **-** |
| *Scrippsiella sp. 1* | Strain | IFR_PALMITO_SBR124 | | NA | NA | LOST | MK660132 (LSU) | Penzé estuary | 14/04/2014 (1998 +/- 4,6) | **X** | **X** | **-** |
| *Scrippsiella donghaienis* | Strain | IFR_PALMITO_SBR128 | | NA | NA | RCC6100 | MK660120 (LSU) | Penzé estuary | 16/05/2014 (1998 +/- 4.6) | **X** | **X** | **-** |
| *Scrippsiella donghaienis* | Strain | IFR_PALMITO_SBR135 | | NA | NA | RCC6115 | MK660128 (LSU) | Penzé estuary | 08/04/2014 (2000 +/- 4.1) | **X** | **X** | **-** |
| *Scrippsiella acuminata* STR1 | Strain | IFR_PALMITO_SBR14 | | NA | NA | RCC6116 | MK660112 (LSU) | Penzé estuary | 24/02/2014 (2006 +/-2.3) | **X** | **X** | **-** |
| *Scrippsiella donghaienis* | Strain | IFR_PALMITO_SBR147 | | NA | NA | RCC6101 | MK660129 (LSU) | Penzé estuary | 05/06/2014 (2000 +/- 4.1) | **X** | **X** | **-** |
| *Scrippsiella lachrymosa* | Strain | IFR_PALMITO_SBR167 | | NA | NA | LOST | MK660134 (LSU) | Brest rade | 16/05/2014 (1993 +/- 1) | **X** | **X** | **-** |
| *Scrippsiella donghaienis* | Strain | IFR_PALMITO_SBR175 | | NA | NA | RCC6117 | MK660130 (LSU) | Penzé estuary | 27/05/2014 (1999 +/- 4,3) | **X** | **X** | **-** |
| *Scrippsiella donghaienis* | Strain | IFR_PALMITO_SBR176 | | NA | NA | RCC6118 | MK660131 (LSU) | Penzé estuary | 27/05/2014 (1999 +/- 4,4) | **X** | **X** | **-** |
| *Scrippsiella donghaienis* | Strain | IFR_PALMITO_SBR177 | | NA | NA | RCC6102 | MK660127 (LSU) | Penzé estuary | 27/05/2014 (1998 +/- 4.6) | **X** | **X** | **-** |
| *Scrippsiella donghaienis* | Strain | IFR_PALMITO_SBR19 | | NA | NA | RCC6103 | MK660123 (LSU) | Penzé estuary | 24/02/2014 (2006 +/-2.3) | **X** | **X** | **-** |
| *Scrippsiella donghaienis* | Strain | IFR_PALMITO_SBR2 | | NA | NA | RCC6104 | MK660121 (LSU) | Penzé estuary | 24/02/2014 (2000 +/- 4.1) | **X** | **X** | **-** |
| *Scrippsiella donghaienis* | Strain | IFR_PALMITO_SBR23 | | NA | NA | RCC6105 | MK660122 (LSU) | Penzé estuary | 28/02/2014 (2006 +/-2.3) | **X** | **X** | **-** |
| *Scrippsiella acuminata* STR1 | Strain | IFR_PALMITO_SBR25 | | NA | NA | RCC6106 | MK660113 (LSU) | Penzé estuary | 28/02/2014 (2006 +/-2.3) | **X** | **X** | **-** |
| *Scrippsiella donghaienis* | Strain | IFR_PALMITO_SBR31 | | NA | NA | RCC6107 | MK660124 (LSU) | Penzé estuary | 28/02/2014 (2002 +/- 3.5) | **X** | **X** | **-** |
| *Scrippsiella donghaienis* | Strain | IFR_PALMITO_SBR4 | | NA | NA | RCC6108 | MK660117 (LSU) | Penzé estuary | 24/02/2014 (2000 +/- 4.1) | **X** | **X** | **-** |
| *Scrippsiella donghaienis* | Strain | IFR_PALMITO_SBR43 | | NA | NA | RCC6109 | MK660118 (LSU) | Penzé estuary | 28/02/2014 (2002 +/- 3.5) | **X** | **X** | **-** |
| *Scrippsiella donghaienis* | Strain | IFR_PALMITO_SBR45 | | NA | NA | RCC6110 | MK660119 (LSU) | Penzé estuary | 28/02/2014 (2002 +/- 3.5) | **X** | **X** | **-** |
| *Scrippsiella lachrymosa* | Strain | IFR_PALMITO_SBR6 | | NA | NA | LOST | MK660135 (LSU) | Penzé estuary | 18/03/2014 (2006 +/- 2,3) | **X** | **X** | **-** |
| *Scrippsiella donghaienis* | Strain | IFR_PALMITO_SBR65 | | NA | NA | RCC6111 | MK660126 (LSU) | Penzé estuary | 03/03/2014 (2006 +/-2.3) | **X** | **X** | **-** |
| Species | Mode | ID_strain/SC | Host_during isolation | | Host_present | Roscoff Culture Collection (RCC) | GenBank acc. Number | Origin | Date of isolation (datation of sediment) | **Use for r**ibotyping | **Use for c**ross-infection | **Use for w**hole genome **sequencing** |
| *-Scrippsiella donghaienis* | Strain | IFR_PALMITO_SBR7 | | NA | NA | RCC6112 | MK660125 (LSU) | Penzé estuary | 24/02/2014 (2000 +/- 4.1) | **X** | **X** | **-** |
| *Scrippsiella donghaienis* | Strain | IFR_PALMITO_SC15 | | NA | NA | RCC4734 | KX009626 (LSU) | Brest rade | 24/02/2014 (1986 +/- 2) | **X** | **X** | **-** |
| *Scrippsiella donghaienis* | Strain | IFR_PALMITO_SC2 | | NA | NA | RCC4733 | KX009636 (LSU) | Brest rade | 24/02/2014 (1991 +/- 1) | **X** | **X** | **-** |
| *Scrippsiella donghaienis* | Strain | IFR_PALMITO_SC20 | | NA | NA | RCC4722 | KX009621 (LSU) | Brest rade | 24/02/2014 (1986 +/- 2) | **X** | **X** | **-** |
| *Scrippsiella donghaienis* | Strain | IFR_PALMITO_SC24 | | NA | NA | RCC4715 | KX009617 (LSU) | Brest rade | 28/02/2014 (1995 +/- 1) | **X** | **X** | **-** |
| *Scrippsiella donghaienis* | Strain | IFR_PALMITO_SC25 | | NA | NA | RCC4716 | KX009616 (LSU) | Brest rade | 28/02/2014 (1995 +/- 1) | **X** | **X** | **-** |
| *Scrippsiella donghaienis* | Strain | IFR_PALMITO_SC27 | | NA | NA | RCC4723 | KX009614 (LSU) | Brest rade | 28/02/2014 (1978 +/- 2) | **X** | **X** | **-** |
| *Scrippsiella donghaienis* | Strain | IFR_PALMITO_SC31 | | NA | NA | RCC4726 | KX009610 (LSU) | Brest rade | 28/02/2014 (2002 +/-3.5) | **X** | **X** | **-** |
| *Scrippsiella acuminata* STR2_Type2 | Strain | IFR_PALMITO_SC32 | | NA | NA | RCC6120 | KX009609 (LSU) | Brest rade | 28/02/2014 (2003 +/-1) | **X** | **X** | **-** |
| *Scrippsiella donghaienis* | Strain | IFR_PALMITO_SC34 | | NA | NA | RCC4711 | KX009607 (LSU) | Brest rade | 28/02/2014 (2010 +/-1) | **X** | **X** | **-** |
| *Scrippsiella donghaienis* | Strain | IFR_PALMITO_SC35 | | NA | NA | RCC4712 | KX009606 (LSU) | Brest rade | 28/02/2014 (2010 +/-1) | **X** | **X** | **-** |
| *Scrippsiella acuminata* STR2_Type2 | Strain | IFR_PALMITO_SC37 | | NA | NA | RCC4732 | KX009604 (LSU) | Brest rade | 28/02/2014 (2006 +/-1) | **X** | **X** | **-** |
| *Scrippsiella donghaienis* | Strain | IFR_PALMITO_SC38 | | NA | NA | RCC4713 | KX009603 (LSU) | Brest rade | 28/02/2014 (2006 +/-1) | **X** | **X** | **-** |
| *Scrippsiella donghaienis* | Strain | IFR_PALMITO_SC4 | | NA | NA | RCC6119 | KX009634 (LSU) | Brest rade | 24/02/2014 (1991 +/- 1) | **X** | **X** | **-** |
| *Scrippsiella acuminata* STR2_Type1 | Strain | IFR_PALMITO_SC45 | | NA | NA | RCC4728 | KX009597 (LSU) | Brest rade | 28/02/2014 (2006 +/-1) | **X** | **X** | **-** |
| *Scrippsiella acuminata* STR2_Type1 | Strain | IFR_PALMITO_SC47 | | NA | NA | RCC6121 | KX009595 (LSU) | Brest rade | 28/02/2014 (2001 +/-1) | **X** | **X** | **-** |
| *Scrippsiella acuminata* STR2_Type1 | Strain | IFR_PALMITO_SC49 | | NA | NA | RCC4729 | KX009593 (LSU) | Brest rade | 28/02/2014 (1997 +/- 1) | **X** | **X** | **-** |
| Amoebophrya RIB5 | Single cell | SC1 (PZ10_12) | | Heterocaspa triquetra | NA | NA | XXXXXXXXX (ITS1, 5.8S, ITS2) | Penzé estuary | 17/06/2010 | **X** | - | - |
| Species | Mode | ID_strain/SC | Host_during isolation | | Host_present | Roscoff Culture Collection (RCC) | GenBank acc. Number | Origin | Date of isolation (datation of sediment) | **Use for r**ibotyping | **Use for c**ross-infection | **Use for w**hole genome **sequencing** |
| Amoebophrya RIB8 | Single cell | SC2 (PZ10_16) | | Scrippsielloid | NA | NA | XXXXXXXXX (ITS1, 5.8S, ITS2) | Penzé estuary | 24/06/2010 | **X** | - | - |
| Amoebophrya RIB8 | Single cell | SC3 (PZ11_16) | | Scrippsielloid | NA | NA | XXXXXXXXX (18S, ITS1-5.8S-ITS2) | Penzé estuary | 08/06/2011 | **X** | - | **X** |
| Amoebophrya RIB8 | Single cell | SC4 (PZ10_18) | | Scrippsielloid | NA | NA | XXXXXXXXX (18S, ITS1-5.8S-ITS2) | Penzé estuary | 24/06/2010 | **X** | - | **X** |
| Amoebophrya RIB8 | Single cell | SC5 (PZ11_20) | | Scrippsielloid | NA | NA | XXXXXXXXX (ITS1, 5.8S, ITS2) | Penzé estuary | 08/06/2011 | **X** | - | - |
| Amoebophrya RIB7 | Single cell | SC6 (RC11_21) | | Scrippsielloid | NA | NA | XXXXXXXXX (ITS1, 5.8S, ITS2) | Rance estuary | 04/06/2011 | **X** | - | **X** |
| Amoebophrya RIB8 | Single cell | SC7 (PZ11_21) | | Scrippsielloid | NA | NA | XXXXXXXXX (ITS1, 5.8S, ITS2) | Penzé estuary | 08/06/2011 | **X** | - | - |
| Amoebophrya RIB8 | Single cell | SC8 (PZ11_22) | | Scrippsielloid | NA | NA | XXXXXXXXX (18S, ITS1-5.8S-ITS2) | Penzé estuary | 08/06/2011 | **X** | - | **X** |
| Amoebophrya RIB6 | Single cell | SC9 (RC11_23) | | Scrippsielloid | NA | NA | XXXXXXXXX (ITS1, 5.8S, ITS2) | Rance estuary | 04/06/2011 | **X** | - | - |
| Amoebophrya RIB8 | Single cell | SC10 (PZ11_23) | | Scrippsielloid | NA | NA | XXXXXXXXX (18S, ITS1-5.8S-ITS2) | Penzé estuary | 08/06/2011 | **X** | - | **X** |
| Amoebophrya RIB6 | Single cell | SC11 (RC11_26) | | Scrippsielloid | NA | NA | XXXXXXXXX (ITS1, 5.8S, ITS2) | Rance estuary | 05/06/2011 | **X** | - | - |
| Amoebophrya RIB5 | Single cell | SC12 (RC11_27) | | Scrippsielloid | NA | NA | XXXXXXXXX (ITS1, 5.8S, ITS2) | Rance estuary | 05/06/2011 | **X** | - | - |
| Amoebophrya RIB8 | Single cell | SC13 (PZ11_27) | | Heterocapsa triquetra | NA | NA | XXXXXXXXX (ITS1, 5.8S, ITS2) | Penzé estuary | 11/06/2011 | **X** | - | - |
| Amoebophrya RIB8 | Single cell | SC14 (RC11_28) | | Scrippsielloid | NA | NA | XXXXXXXXX (18S, ITS1-5.8S-ITS2) | Rance estuary | 05/06/2011 | **X** | - | **X** |
| Amoebophrya RIB8 | Single cell | SC15 (PZ11_29) | | Scrippsielloid | NA | NA | XXXXXXXXX (18S, ITS1-5.8S-ITS2) | Penzé estuary | 11/06/2011 | **X** | - | **X** |
| Amoebophrya RIB6 | Single cell | SC16 (PZ11_3) | | Scrippsielloid | NA | NA | XXXXXXXXX (ITS1, 5.8S, ITS2) | Penzé estuary | 06/06/2011 | **X** | - | - |
| Amoebophrya RIB8 | Single cell | SC17 (RC11_30) | | Scrippsielloid | NA | NA | XXXXXXXXX (ITS1, 5.8S, ITS2) | Rance estuary | 05/06/2011 | **X** | - | - |
| Amoebophrya RIB5 | Single cell | SC18 (RC11_31) | | Scrippsielloid | NA | NA | XXXXXXXXX (ITS1, 5.8S, ITS2) | Rance estuary | 05/06/2011 | **X** | - | - |
| Amoebophrya RIB8 | Single cell | SC19 (PZ11_36) | | Heterocapsa triquetra | NA | NA | XXXXXXXXX (ITS1, 5.8S, ITS2) | Penzé estuary | 11/06/2011 | **X** | - | - |
| Amoebophrya RIB2 | Single cell | SC20 (PZ11_39) | | Heterocapsa triquetra | NA | NA | XXXXXXXXX (ITS1, 5.8S, ITS2) | Penzé estuary | 11/06/2011 | **X** | - | - |
| Species | Mode | ID_strain/SC | Host_during isolation | | Host_present | Roscoff Culture Collection (RCC) | GenBank acc. Number | Origin | Date of isolation (datation of sediment) | **Use for r**ibotyping | **Use for c**ross-infection | **Use for w**hole genome **sequencing** |
| Amoebophrya RIB1 | Single cell | SC21 (PZ11_4) | | Scrippsielloid | NA | NA | XXXXXXXXX (ITS1, 5.8S, ITS2) | Penzé estuary | 06/06/2011 | **X** | - | - |
| Amoebophrya RIB8 | Single cell | SC22 (RC11_4) | | Scrippsielloid | NA | NA | XXXXXXXXX (ITS1, 5.8S, ITS2) | Rance estuary | 28/05/2011 | **X** | - | - |
| Amoebophrya RIB6 | Single cell | SC23 (RC11_41) | | Scrippsielloid | NA | NA | XXXXXXXXX (ITS1, 5.8S, ITS2) | Rance estuary | 09/06/2011 | **X** | - | - |
| Amoebophrya RIB8 | Single cell | SC24 (PZ11_41) | | Scrippsielloid | NA | NA | XXXXXXXXX (ITS1, 5.8S, ITS2) | Penzé estuary | 14/06/2011 | **X** | - | - |
| Amoebophrya RIB8 | Single cell | SC25 (PZ11_42) | | Scrippsielloid | NA | NA | XXXXXXXXX (18S,ITS1-5.8S-ITS2) | Penzé estuary | 14/06/2011 | **X** | - | **X** |
| Amoebophrya RIB6 | Single cell | SC26 (PZ11_45) | | Scrippsielloid | NA | NA | XXXXXXXXX (ITS1, 5.8S, ITS2) | Penzé estuary | 14/06/2011 | **X** | - | - |
| Amoebophrya RIB3 | Single cell | SC27 (PZ11_46) | | Scrippsielloid | NA | NA | XXXXXXXXX (ITS1, 5.8S, ITS2) | Penzé estuary | 14/06/2011 | **X** | - | - |
| Amoebophrya RIB8 | Single cell | SC28 (RC11_5) | | Scrippsielloid | NA | NA | XXXXXXXXX (ITS1, 5.8S, ITS2) | Rance estuary | 28/05/2011 | **X** | - | - |
| Amoebophrya RIB2 | Single cell | SC29 (PZ11_50) | | Scrippsielloid | NA | NA | XXXXXXXXX (ITS1, 5.8S, ITS2) | Penzé estuary | 14/06/2011 | **X** | - | - |
| Amoebophrya RIB4 | Single cell | SC30 (PZ11_51) | | Scrippsielloid | NA | NA | XXXXXXXXX (ITS1, 5.8S, ITS2) | Penzé estuary | 14/06/2011 | **X** | - | - |
| Amoebophrya RIB8 | Single cell | SC31 (PZ11_53) | | Scrippsielloid | NA | NA | XXXXXXXXX (ITS1, 5.8S, ITS2) | Penzé estuary | 14/06/2011 | **X** | - | - |
| Amoebophrya RIB4 | Single cell | SC32 (PZ11_54) | | Heterocapsa triquetra | NA | NA | XXXXXXXXX (ITS1, 5.8S, ITS2) | Penzé estuary | 14/06/2011 | **X** | - | - |
| Amoebophrya RIB2 | Single cell | SC33 (PZ11_55) | | Heterocapsa triquetra | NA | NA | XXXXXXXXX (ITS1, 5.8S, ITS2) | Penzé estuary | 14/06/2011 | **X** | - | **X** |
| Amoebophrya RIB8 | Single cell | SC34 (PZ11_56) | | Heterocapsa triquetra | NA | NA | XXXXXXXXX (ITS1, 5.8S, ITS2) | Penzé estuary | 14/06/2011 | **X** | - | - |
| Amoebophrya RIB3 | Single cell | SC35 (PZ11_58) | | Scrippsielloid | NA | NA | XXXXXXXXX (ITS1, 5.8S, ITS2) | Penzé estuary | 14/06/2011 | **X** | - | **X** |
| Amoebophrya RIB2 | Single cell | SC36 (PZ11_59) | | Scrippsielloid | NA | NA | XXXXXXXXX (ITS1, 5.8S, ITS2) | Penzé estuary | 14/06/2011 | **X** | - | - |
| Amoebophrya RIB2 | Single cell | SC37 (PZ11_60) | | Scrippsielloid | NA | NA | XXXXXXXXX (ITS1, 5.8S, ITS2) | Penzé estuary | 14/06/2011 | **X** | - | **X** |
| Amoebophrya RIB8 | Single cell | SC38 (PZ11_61) | | Scrippsielloid | NA | NA | XXXXXXXXX (18S, ITS1-5.8S-ITS2) | Penzé estuary | 17/06/2011 | **X** | - | **X** |
| Amoebophrya RIB5 | Single cell | SC39 (PZ11_62) | | Scrippsielloid | NA | NA | XXXXXXXXX (ITS1, 5.8S, ITS2) | Penzé estuary | 17/06/2011 | **X** | - | - |
| Species | Mode | ID_strain/SC | Host_during isolation | | Host_present | Roscoff Culture Collection (RCC) | GenBank acc. Number | Origin | Date of isolation (datation of sediment) | **Use for r**ibotyping | **Use for c**ross-infection | **Use for w**hole genome **sequencing** |
| Amoebophrya RIB3 | Single cell | SC40 (PZ11_63) | | Scrippsielloid | NA | NA | XXXXXXXXX (ITS1, 5.8S, ITS2) | Penzé estuary | 17/06/2011 | **X** | - | - |
| Amoebophrya RIB8 | Single cell | SC41 (PZ11_64) | | Scrippsielloid | NA | NA | XXXXXXXXX (18S, ITS1-5.8S-ITS2) | Penzé estuary | 17/06/2011 | **X** | - | **X** |
| Amoebophrya RIB5 | Single cell | SC42 (PZ10_7) | | Heterocaspa triquetra | NA | NA | XXXXXXXXX (ITS1, 5.8S, ITS2) | Penzé estuary | 16/06/2010 | **X** | - | - |
| Amoebophrya RIB8 | Single cell | SC43 (RC11_7) | | Scrippsielloid | NA | NA | XXXXXXXXX (ITS1, 5.8S, ITS2) | Rance estuary | 28/05/2011 | **X** | - | - |
| *Scrippsiella acuminata* STR2_Type2 | Strain |  | | NA | NA | RCC1720 | MK674084 (LSU) | SOMLIT ASTAN | 13/05/2008 | **X** | **X** | **-** |

**Table S2: Statistic of the genome assemblies: read number (total and after filtration), N50 (Total and > 1000 kb), remaping rate of reads and average coverage.**

| Strain or Single cell | Total reads | Filtered reads count | N50 | N50 (>1000bp) | Remapping rate | Average coverage |
| --- | --- | --- | --- | --- | --- | --- |
| A1 | 32 597 788 | 27545422 | 40522 | 41612 | 0.9316 | 41.2111 |
| A24 | 22 170 180 | 15938544 | 26540 | 28275 | 0.9189 | 25.4989 |
| A29 | 65979782 | 52436534 | 47986 | 48999 | 0.9561 | 85.8073 |
| A30 | 51615038 | 40469950 | 47467 | 48447 | 0.9561 | 67.6153 |
| A32 | 40 027 570 | 31811814 | 47946 | 49352 | 0.9373 | 47.0513 |
| A33 | 53163770 | 42648628 | 46543 | 48069 | 0.954 | 69.5386 |
| A34 | 70966608 | 58662234 | 38412 | 40462 | 0.9504 | 88.9921 |
| A37 | 64406860 | 53939788 | 23421 | 24880 | 0.9552 | 80.2298 |
| A42 | 41 309 688 | 37943270 | 16486 | 17556 | 0.9009 | 34.3543 |
| A46 | 30 299 444 | 26409052 | 18302 | 18900 | 0.9378 | 38.527 |
| A48 | 53343078 | 45069552 | 23195 | 23880 | 0.9525 | 69.1362 |
| A49 | 73049494 | 57191260 | 49348 | 52032 | 0.9554 | 92.5387 |
| A51 | 56891848 | 45454372 | 42243 | 48507 | 0.9438 | 67.2662 |
| A54 | 41345204 | 31884946 | 34790 | 36036 | 0.9373 | 64.1275 |
| A71 | 45778458 | 41698404 | 16451 | 17719 | 0.9199 | 38.1075 |
| A72 | 32 203 188 | 29695764 | 14883 | 15657 | 0.8859 | 26.4288 |
| A74 | 40 360 306 | 16201658 | 7055 | 8488 | 0.9266 | 27.0515 |
| A75 | 56756818 | 45081158 | 47672 | 49291 | 0.9542 | 73.9866 |
| A76 | 28 310 694 | 26250730 | 16308 | 16969 | 0.9003 | 23.5293 |
| A78 | 63266228 | 20037572 | 19235 | 23889 | 0.9294 | 67.8865 |
| A91 | 49042824 | 43141252 | 15147 | 19085 | 0.8651 | 49.2374 |
| A97 | 46 036 804 | 39440992 | 22806 | 23423 | 0.9461 | 58.8624 |
| A98 | 46 570 476 | 42506420 | 16426 | 17535 | 0.9091 | 37.7321 |
| A99 | 42878088 | 39009314 | 16915 | 17947 | 0.9156 | 36.1088 |
| A100 | 66401074 | 59685208 | 17822 | 18875 | 0.9216 | 54.6545 |
| A103 | 57958210 | 52046724 | 18496 | 19384 | 0.9237 | 49.3881 |
| A106 | 57259114 | 51905410 | 18289 | 19244 | 0.9201 | 48.6952 |
| Strain or Single cell | Total reads | Filtered reads count | N50 | N50 (>1000bp) | Remapping rate | Average coverage |
| A107 | 56306672 | 52267272 | 9044 | 10905 | 0.8852 | 42.0832 |
| A110 | 40100050 | 32197968 | 38645 | 42651 | 0.9383 | 45.1752 |
| A111 | 61902422 | 56453154 | 16001 | 18152 | 0.9122 | 48.8841 |
| A114 | 54334936 | 49506978 | 14849 | 16764 | 0.9161 | 41.067 |
| A117 | 52766046 | 48775460 | 12750 | 14522 | 0.9006 | 41.2465 |
| A126 | 52654516 | 48383588 | 14574 | 16522 | 0.9032 | 41.9721 |
| A127 | 43 000 160 | 39483346 | 16318 | 17649 | 0.9051 | 34.7821 |
| A129 | 38 361 080 | 33170086 | 20514 | 21069 | 0.9441 | 49.2397 |
| A135 | 41 192 256 | 37716018 | 21052 | 21710 | 0.9081 | 34.9305 |
| A136 | 44 945 232 | 40804012 | 17800 | 18824 | 0.9004 | 38.0065 |
| A137 | 68183234 | 62126772 | 16138 | 17436 | 0.9026 | 55.786 |
| A138 | 61799720 | 55524646 | 18723 | 19606 | 0.922 | 53.5879 |
| A139 | 39 978 988 | 36850220 | 18178 | 18919 | 0.9057 | 33.5654 |
| A141 | 58564830 | 51076030 | 16969 | 19812 | 0.9251 | 42.9193 |
| A142 | 43673922 | 39927002 | 19750 | 20451 | 0.9231 | 37.511 |
| A144 | 42098046 | 38433110 | 17111 | 18065 | 0.9162 | 36.6419 |
| A146 | 42 121 926 | 38857576 | 17072 | 18094 | 0.8963 | 35.2477 |
| A147 | 49472368 | 39955422 | 30574 | 33333 | 0.922 | 33.1948 |
| A149 | 49604200 | 40403686 | 30784 | 34095 | 0.9219 | 32.9939 |
| A150 | 70304300 | 58422836 | 22880 | 25568 | 0.9319 | 95.1714 |
| A151 | 39 005 356 | 6542732 | 3123 | 12835 | 0.9469 | 20.1562 |
| A152 | 57278004 | 53076884 | 13648 | 15424 | 0.9044 | 46.579 |
| A154 | 71473328 | 55056178 | 36810 | 38011 | 0.9433 | 51.4394 |
| PZ10_SC18 | 31961110 | 3038564 | 3429 | 5790 | 0.9587 | 46.4045 |
| PZ11_SC16 | 33792376 | 18817268 | 2420 | 3793 | 0.9156 | 128.088 |
| PZ11_SC20 | 34209206 | 26460326 | 1520 | 2926 | 0.5371 | 300.497 |
| PZ11_SC22 | 34306130 | 29688056 | 2688 | 4088 | 0.8808 | 37.6085 |
| PZ11_Sc23 | 33718280 | 30468090 | 2290 | 3486 | 0.8777 | 181.152 |
| PZ11_SC29 | 33697940 | 31584928 | 4370 | 5568 | 0.9336 | 55.9334 |
| Strain or Single cell | Total reads | Filtered reads count | N50 | N50 (>1000bp) | Remapping rate | Average coverage |
| PZ11_SC41 | 49321478 | 46204634 | 1540 | 3407 | 0.8537 | 141.487 |
| PZ11_SC42 | 48094994 | 40317924 | 5334 | 6703 | 0.9074 | 59.5274 |
| PZ11_SC55 | 33797508 | 29474132 | 4631 | 6693 | 0.851 | 74.1605 |
| PZ11_SC58 | 33633210 | 25078280 | 2495 | 3674 | 0.9368 | 128.145 |
| PZ11_SC60 | 34306696 | 31837950 | 2311 | 3757 | 0.8637 | 89.619 |
| PZ11_SC61 | 33555166 | 30037224 | 3230 | 4638 | 0.9487 | 136.417 |
| PZ11_SC64 | 33404478 | 27752464 | 3225 | 4660 | 0.924 | 68.2133 |
| RC11_SC21 | 34943860 | 17228812 | 1699 | 4692 | 0.8715 | 88.4824 |
| RC11_SC28 | 37527660 | 35263616 | 1906 | 3851 | 0.5883 | 106.311 |
| RC11_SC30 | 42337260 | 41869542 | 1985 | 3339 | 0.5277 | 245.002 |
| RC11_SC5 | 29459360 | 13402234 | 1363 | 2901 | 0.8861 | 482.56 |

**Table S3: Nucleotide variability (top right) and percent identity (bottom left) of the complete SSU rDNA gene (top in each cell), 18S-V4 (middle in each cell) and 18S-V9 (bottom in each cell) regions between the eight ribotypes defined in this study.**
